# Non-Target Effects of dsRNA Molecules in Hemipteran Insects

**DOI:** 10.3390/genes12030407

**Published:** 2021-03-12

**Authors:** Arinder K. Arora, Seung Ho Chung, Angela E. Douglas

**Affiliations:** 1 Department of Entomology, Cornell University, Ithaca, NY 14850, USA; sc776@cornell.edu (S.H.C.); aes326@cornell.edu (A.E.D.); 2 Department of Molecular Biology and Genetics, Cornell University, Ithaca, NY 14853, USA

**Keywords:** *Acyrthosiphon pisum*, *Bemisia tabaci*, *Myzus persicae*, sequence-non-specific RNAi, non-target organisms, nucleases of insects, plant sap-feeding insects, *Pseudococcus maritimus*, RNAi specificity

## Abstract

Insect pest control by RNA interference (RNAi)-mediated gene expression knockdown can be undermined by many factors, including small sequence differences between double-stranded RNA (dsRNA) and the target gene. It can also be compromised by effects that are independent of the dsRNA sequence on non-target organisms (known as sequence-non-specific effects). This study investigated the species-specificity of RNAi in plant sap-feeding hemipteran pests. We first demonstrated sequence-non-specific suppression of aphid feeding by dsRNA at dietary concentrations ≥0.5 µg µL^−1^. Then we quantified the expression of *NUC* (nuclease) genes in insects administered homologous dsRNA (with perfect sequence identity to the target species) or heterologous dsRNA (generated against a related gene of non-identical sequence in a different insect species). For the aphids *Acyrthosiphon pisum* and *Myzus persicae*, significantly reduced *NUC* expression was obtained with the homologous but not heterologous dsRNA at 0.2 µg µL^−1^, despite high ds*NUC* sequence identity. Follow-up experiments demonstrated significantly reduced expression of *NUC* genes in the whitefly *Bemisia tabaci* and mealybug *Planococcus maritimus* administered homologous *dsNUCs*, but not heterologous aphid ds*NUC*s. Our demonstration of inefficient expression knockdown by heterologous dsRNA in these insects suggests that maximal dsRNA sequence identity is required for RNAi targeting of related pest species, and that heterologous dsRNAs at appropriate concentrations may not be a major risk to non-target sap-feeding hemipterans.

## 1. Introduction

Ground-breaking studies on corn root worm and cotton bollworm conducted over a decade ago [1,2] provided proof-of-concept for the use of RNA interference (RNAi) in insect pest control. Since then, major advances have been made to understand the mechanism of RNAi in insects; to document among-species variation in RNAi efficacy; and to elucidate the genetic, molecular and biochemical bases of barriers to RNAi-triggered gene silencing [3,4,5]. RNAi, unlike most traditional insect control technologies, offers the potential for exquisite control over the specificity of control agents [6,7]. This is because the enzyme of the *Argonaute* family in the RNA-induced silencing complex (RISC) is a guide-dependent RNase, requiring perfect sequence complementarity between the target transcript and the 21 nt guide RNA, a small interfering RNA (siRNA) generated by Dicer-mediated cleavage of double-stranded RNA (dsRNA) [8]. There is a general expectation that by careful selection of the length and sequence of dsRNA, RNAi can be limited to target insect pest(s) without any direct, deleterious effect on non-target species, including beneficial insects such as pollinators and biological control agents [9,10].

The motivation for our research was the increasing recognition that empirical studies on the taxon specificity of RNAi are essential for the successful application of this technology to insect pest control. Our experimental design investigated the gene silencing mediated by two types of dsRNA sequence administered to an insect species: a dsRNA with perfect sequence identity to the gene in that species (homologous dsRNA) and a dsRNA generated against a related gene of non-identical sequence in a different insect species (heterologous dsRNA). The percentage of sequence identity between the homologous and heterologous dsRNA sequences, although frequently used, is an imprecise indicator of the RNAi efficacy of heterologous dsRNA molecules because sequences of the same percentage identity can vary widely in the number of identical 21 nt sequences that trigger transcript degradation [11,12]. Bioinformatic tools are available to calculate the number of 21-mers with or without mismatches in sequenced insect genomes or individual gene transcripts [13,14]. However, these predictions of dsRNA specificity can be confounded by two issues. First, RNAi susceptibility to heterologous dsRNA can be obtained with a single matching 21-mer sequence, require several or many matching 21-mers, be achieved with as few as 15 contiguously matching bases and be tolerant of some sequence mismatches—varying with species, gene and dsRNA concentration [11,15,16,17,18]. Second, animals, including insects, can respond to dsRNA in a sequence-non-specific manner, i.e., independent of the sequence of the dsRNA. In particular, various studies have shown that dsRNA with no matching 15–21-mer sequences in an insect genome can have substantial effects on antiviral immunity, gene expression and performance in insects [16,19,20,21,22,23,24], although the molecular mechanisms are not fully understood. These effects are particularly pronounced for dsRNA administered at high concentrations, and it has been argued that high siRNA titers may saturate the core RNAi machinery [11,25].

The specific aim of this study was to investigate the species specificity of dsRNA effects on gene expression in plant phloem sap-feeding hemipteran insects of the suborder Sternorrhyncha (aphids, whiteflies, scale insects, etc.). This group of insects includes many economically-important crop pests. Our study concerned four pest species within three sternorrhynchan superfamilies: two aphids *Acyrthosiphon pisum* and *Myzus persicae* (both members of the tribe Macrosiphini within superfamily Aphidoidea), the mealybug *Pseudococcus maritimus* (superfamily Coccoidea, sister group of Aphidoidea) and the whitefly *Bemisia tabaci* (in the more distantly related superfamily Aleyrodoidea). In this way, we were able to investigate the responses of insects to dsRNA designed against insects with different degrees of relatedness. All four species are agricultural pests. *B*. *tabaci* and *M*. *persicae* are highly invasive pests of a wide range of crop plants, and they are capable of transmitting more than 150 and 100 plant viruses, respectively [26,27]. *A*. *pisum* is a global pest of legume crops and is reported to transmit more than 30 viruses [28]. *P*. *maritimus* vectors grapevine leafroll-associated viruses (GLRaV) and is a serious threat to grape production in North America [29]. These insects are controlled mainly by chemical insecticides. However, insecticide resistance is widespread in these insects, particularly *M. persicae* and *B*. *tabaci* [30,31,32], and insecticide applications against *P*. *maritimus* have little impact on GLRaV incidence [33]. These observations indicate the need for novel methods to control these insects.

RNAi has great potential for crop protection against sternorrhynchan insect pests, following many demonstrations that the survival and population increase of various species are curtailed by RNAi against various essential genes [34,35,36,37,38]. The *NUC* genes, which code nucleases, are particularly well-suited to this purpose for several reasons. The sequences of the relevant genes are known, and phylogenetic analyses have demonstrated that variation in *NUC* gene sequence between our test species matches to the species taxonomy [39]. *NUC* gene expression in all species is enriched in the gut and it is significantly reduced by orally-delivered homologous ds*NUC* (i.e., dsRNA against the *NUC* gene in the test species) [37,39,40]. Furthermore, the *NUC* genes are not essential genes, facilitating analysis of the effects of heterologous ds*NUCs* (i.e., derived from different species) on *NUC* expression without high insect mortality; and ds*NUC*s are used in RNAi studies to suppress nuclease-mediated degradation of dsRNA administered to these species [37,39,40] and other insects [41,42]. In this study, we found that only homologous ds*NUCs* triggered *NUC* expression knockdown in all four species. These results provide indications that non-target hemipterans in agroecosystems would not be particularly susceptible to heterologous dsRNAs, and that dsRNAs designed against multiple hemipteran pests should include high numbers of 21-mer siRNAs that match the sequences of all the target species.

## 2. Materials and Methods

### 2.1. Insects

*Acyrthosiphon pisum* clone CWR09/18 [43] and clone SC_37 [44], each derived from a single parthenogenetic female collected from alfalfa crop in Freeville, NY and Ithaca, NY (USA), respectively, and were maintained on pre-flowering *Vicia faba* cv. Windsor at 20 °C with 16L:8D light cycle. The New York GPA clone of *Myzus persicae* [38] was reared at 25 °C and 16L:8D light cycle on *Brassica juncea* cv. Florida Broad Leaf. *Bemisia tabaci* MEAM1 derived from a collection from *Euphorbia pulcherrima* Willd. Ex Klotzsch in Ithaca, NY, USA in 1989 was maintained on *Solanum lycopersicum* cv. Florida Lanai at 25 °C and 14L:10D light. A culture of *Pseudococcus maritimus* were generated from three collections from *Vitis vinifera* cv. Chardonnay in Seneca County, NY in July-August, 2018 and maintained on *V*. *vinifera* cv. Pixie at 21 °C with 17L:7D [39].

### 2.2. RNA Extraction

Insects were homogenized in 300 µL RNAzol (catalog number R4533, Millipore Sigma, Burlington, MA) using Lysing matrix D beads (catalog number 116913050, MP Biomedicals, Santa Ana, CA, USA) and a FastPrep homogenizer (Santa Ana, CA, USA). Following addition of 120 µL water, the homogenate was vortexed vigorously for 15 s and incubated at room temperature for 15 min. The mixture was centrifuged at 12,000× *g* and 4 °C for 15 min and 360 µL supernatant was transferred to a new microcentrifuge tube. The supernatant was combined with 1.8 µL 4-bromoanisole and vortexed vigorously for 15 s followed by incubation at room temperatures for 5 min, and centrifugation at 12,000× *g* and 4 °C for 10 min. The supernatant was transferred to a clean microcentrifuge tube and an equal amount of isopropanol and 1 µL linear acrylamide was added and mixed by vortexing. The mixture was incubated for 10 min at room temperature for the aphids, and overnight at −20 °C for whiteflies and mealybugs. Following incubation, the samples were centrifuged, and the RNA pellet was washed twice with 75% alcohol and suspended in 25 µL nuclease-free water. The RNA was free of genomic DNA, as indicated by the absence of detectable product in qPCR assays of cDNA samples generated with no reverse transcriptase (using primers predicted to yield the same product for cDNA and genomic DNA).

### 2.3. cDNA Synthesis

cDNA was synthesized from 500 ng RNA in a 20 µL reaction using Superscript™ II kit (catalog number 18064014, ThermoFisher Scientific, Waltham, MA, USA). A 12 µL reaction containing 1 µL random primers, 1 µL dNTPs and 500 ng RNA was incubated at 65 °C for 5 min and transferred to ice. Two µL dithiothreitol, 4 µL 5× buffer and 1 µL water were added to the mixture and incubated at 25 °C for 2 min, followed by a transfer to ice, and addition of 1 µL reverse transcriptase. The final reaction was incubated at 25 °C for 10 min, 42 °C for 50 min and 70 °C for 15 min. The cDNA was stored at −20 °C.

### 2.4. Quantitative Real Time-PCR (qRT-PCR)

The 10 µL reaction mixtures were run in a C1000 Thermal Cycler with CFX96 TouchTM Real-Time PCR Detection System (Bio-Rad, Hercules, CA, USA). The reaction mixture contained 5 µL iQ SYBR Green supermix (catalog number 1708862, Bio-Rad), 1 µL cDNA, 3 µL water and 0.5 µM forward and reverse primers (Supplementary Table S1A). The template cDNA was dissociated at 95 °C for 3 min followed by 40 cycles of 95 °C for 10 s and 60 °C for 30 s. A dissociation curve, from 65 °C to 95 °C in 0.5 °C increment per 0.05 s, was generated for every sample and confirmed the specificity of the PCR reactions. *β-tubulin* and RPL32 were used as reference genes for *A*. *pisum* and *M*. *persicae*, and *β-tubulin* was used as reference gene for *B*. *tabaci* and *P*. *maritimus*, following published protocols [37,39,40]. No-template reaction was used as negative control. Average Ct-values of two technical replicates were calculated and the difference between treatment delta Ct and control delta Ct was used to calculate fold changes [45].

### 2.5. dsRNA Synthesis

Target sequences of *NUC* genes were amplified using primers (Supplementary Table S1B) with the T7 promoter sequence at the 5′ end of both forward and reverse primers and cloned in pGEMT Easy plasmid (catalog number A1360, Promega, Madison, WI, USA). The plasmid was transformed into *Escherichia coli* DH5α (catalog number 18258012, ThermoFisher Scientific) using the heat shock method. The ds*GFP* template comprised a 370 bp fragment of the *GFP* gene (coding the green fluorescent protein of the jellyfish *Aequorea victoria*) previously cloned into pGFP2 plasmid in *E*. *coli* DH5α, as in [37,40]. The dsRNA was purified with the Zymo DNA Clean and Concentrator kit (catalog number D4033, Irvine, CA, USA) following the manufacturer’s protocol, and then quantified using a Nanodrop spectrophotometer.

A 20 µL reaction with 1 µg template DNA was used to synthesize dsRNA using AmpliScribe T7-Flash Transcription kit (catalog number ASF3507, Lucigen, Middleton, WI, USA). The template DNA was then removed by addition of 1µL RNase-free DNase at the end of the reaction. To remove the NTPs and other impurities, 79 µL water was added for a final volume of 100 µL, followed by the addition of an equal volume of 5 M ammonium acetate (catalog number AM9070G, ThermoFisher Scientific) and 250 µL ethanol. The mixture was incubated at −20 °C overnight, and then centrifuged at 12,000× *g* at 4 °C for 20 min. The resulting RNA pellet was washed twice with 75% ethanol and resuspended in 30 µL nuclease-free water.

### 2.6. Experimental Designs

For all experiments, the insects were administered dsRNA over two days by feeding from a liquid artificial diet aseptically enclosed within stretched Parafilm sheets, as described previously [46]. The diet comprised 0.5 M sucrose, amino acids at a total concentration of 0.15 M, micronutrients with potassium phosphate buffer, pH 7.0 [46], and dsRNA is stable in this diet [37,40]. For each species, all diet experiments were conducted under the same temperature and light regime as used for the plant-reared insects. The experiments on aphids included a pre-treatment of 2-to-5-day-old nymphs on dsRNA-free diets because *A*. *pisum* reared on plants to ages > 2 days do not feed well from diets.

**Figure 1:**
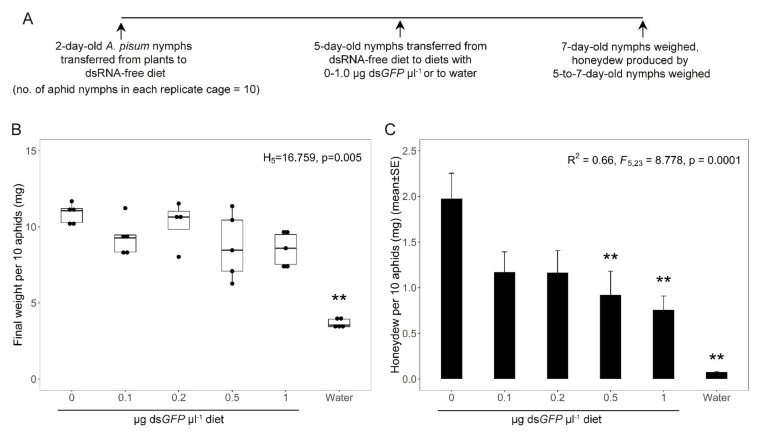
Response of *A. pisum* CWR09/18 to dietary ds*GFP*, administered to five replicate groups of ten 5-day-old nymphs for two days. (**A**) Experimental design. (**B**) Total weight of the ten 7-day-old nymphs per cage. (**C**) Honeydew production by the ten aphids per cage over the two-day experiment. Double asterisks identify treatments are significantly different from ds*GFP*-free diet at α = 0.01. Five replicate cages were assayed for all treatments, except 0.2 μg µL^−1^, with four replicate cages.

The first experiment quantified food consumption of *A*. *pisum* clone CWR09/18 administered ds*GFP*. Thirty replicate groups of ten 2-day-old plant-reared nymphs were administered dsRNA-free artificial diet [46] (Figure 1A). When 5-days-old, five replicate groups of 10 nymphs each were transferred to the artificial liquid diet supplemented with ds*GFP* at each of 0, 0.1, 0.2, 0.5 and 1 µg µL^−1^ or water only in diet cages (3.5 cm diameter × 0.5 cm height). A pre-weighed circle of aluminum foil was placed under the feeding aphids in each diet cage to collect the honeydew. Two days later (day 7), the number of surviving aphids per cage was scored, and the pooled weight determined. The honeydew accumulation was quantified by subtracting the final weight of the aluminum foil from the initial weight. All weights were determined on a Mettler MT5 microbalance to the nearest μg. All the aphids in every diet cage survived the experiment, apart from one replicate containing 0.2 µg µL^−1^ diet, and this replicate was removed from the analysis.

**Figure 2:**
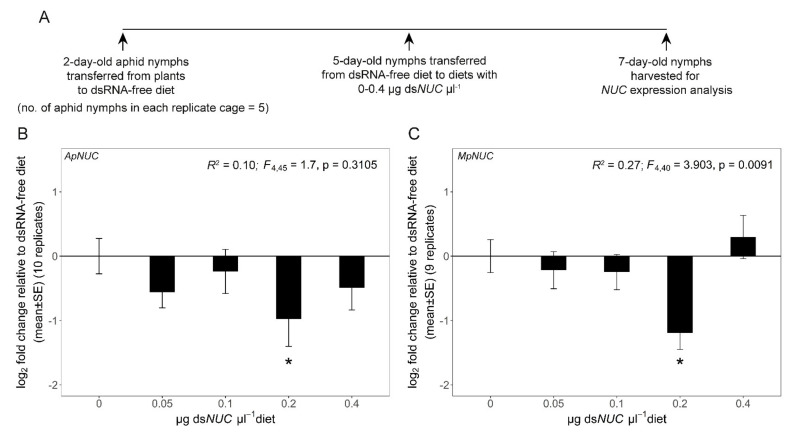
Variation in aphid *NUC* expression with dietary concentration of ds*NUC*. (**A**) Experimental design. (**B**) *A*. *pisum NUC* gene expression. (**C**) *M. persicae NUC* gene expression. Linear model statistics were employed using ds*NUC*- free diet as the intercept to identify the concentrations of dietary ds*NUC* that had a significant effect on *NUC* expression. Asterisks identify treatments significantly different from ds*GFP*-free diet at α = 0.05. The pooled data for two iterations are shown (5 replicate cages for every treatment in the first iteration, and 5 and 4 replicate cages for each treatment for *A*. *pisum* and *M*. *persicae*, respectively, in the second iteration).

The second experiment determined the effect of homologous ds*NUC* on *NUC* expression in aphids. Each replicate group of five 5-day-old *A*. *pisum* that had been raised on dsRNA-free diet from day 2, were administered ds*ApNUC* (dsRNA against the nuclease gene *NUC* of *A*. *pisum* NCBI accession number XM_003242604.4, described in [40] as *NUC1*) at 0, 0.05, 0.1, 0.2 and 0.4 µg µL^−1^ diet (Figure 2A). The same experimental design was adopted for *M*. *persicae*, using ds*MpNUC* (dsRNA against the *M. persicae NUC* gene, NCBI accession number XM_022327343.1) (Figure 2A). The aphids were harvested on day-7 and the group of aphids from each cage (i.e., each replicate) was stored at −80 °C prior to RNA extraction. As the supply of insects was limited, the experiment was conducted twice for each species. In the first iteration, five replicates were used for both species. In the second iteration, five replicates were used for *A*. *pisum* and four for *M*. *persicae*. The data did not differ significantly between the two iterations (*p* = 0.43 for *A*. *pisum* and *p* = 0.90 for *M*. *persicae*), and the results for the two iterations for each species were pooled, yielding 10 and 9 replicate cages for *A. pisum* and *M*. *persicae*, respectively.

**Figure 3:**
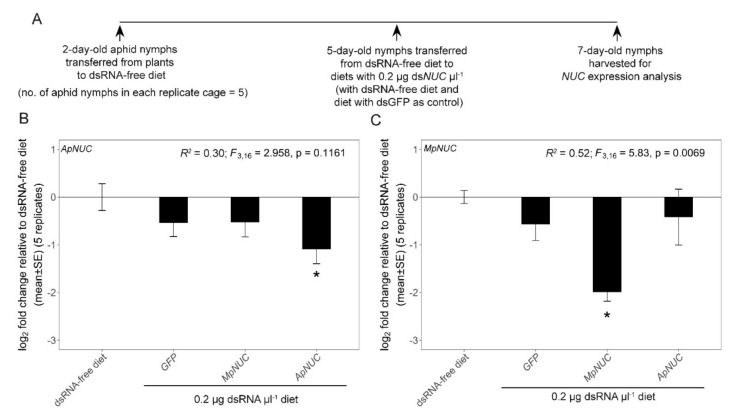
Cross-reactivity of ds*NUC* between aphid species. Fold-difference in *NUC* expression of aphids administered dsRNA at 0.2 µg μL^−1^ in the diet for two days. (**A**) Experimental design. (**B**) *A*. *pisum NUC* gene expression. (**C**) *M*. *persicae NUC* gene expression. The experiment comprised five replicate cages for each treatment. Linear model statistics were employed using dsRNA-free diet as the intercept to identify significant effect of dietary dsRNAs on *NUC* expression. Asterisks identify treatments that were significantly different from the ds*GFP*-free diet at α = 0.05. *ApNUC*, *A. pisum NUC*; *MpNUC*, *M. persicae NUC*.

**Figure 4:**
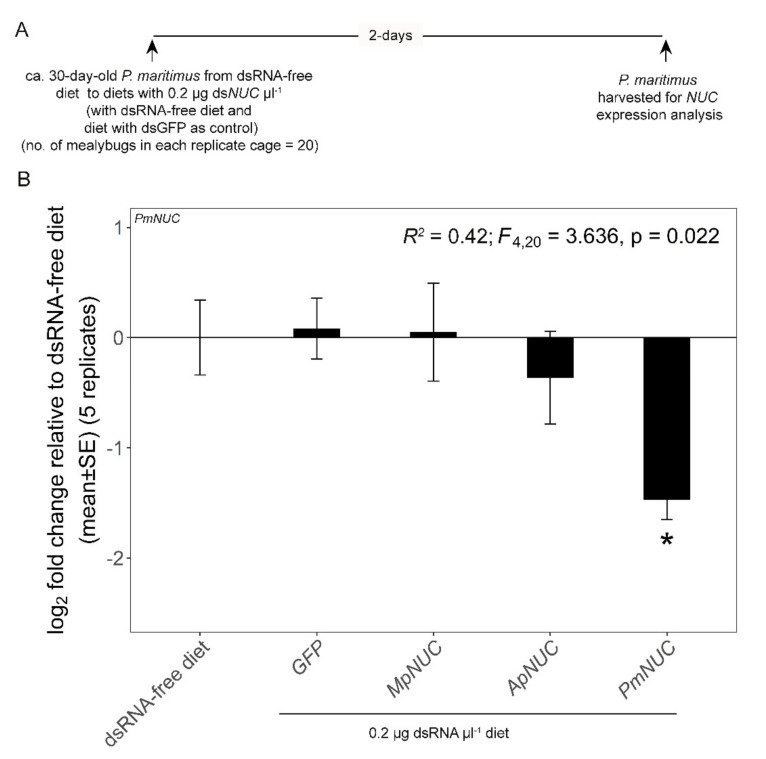
Activity of aphid ds*NUC* against *NUC* gene expression in *P. maritimus*. (**A**) Experimental design. (**B**) *P. maritimus NUC* gene expression. The pooled data for two iterations of the experiment are shown (with three and two replicate cages per treatment in the first and second iterations, respectively). Linear model statistics were employed using dsRNA-free diet as the intercept to identify significant effects of various dietary dsRNAs on *NUC* expression. Asterisks identify treatments that are significantly different from dsGFP-free diet at α = 0.05. *ApNUC*, *A. pisum NUC*; *MpNUC*, *M. persicae NUC*; *PmNUC*, *P*. *maritimus NUC*.

**Figure 5:**
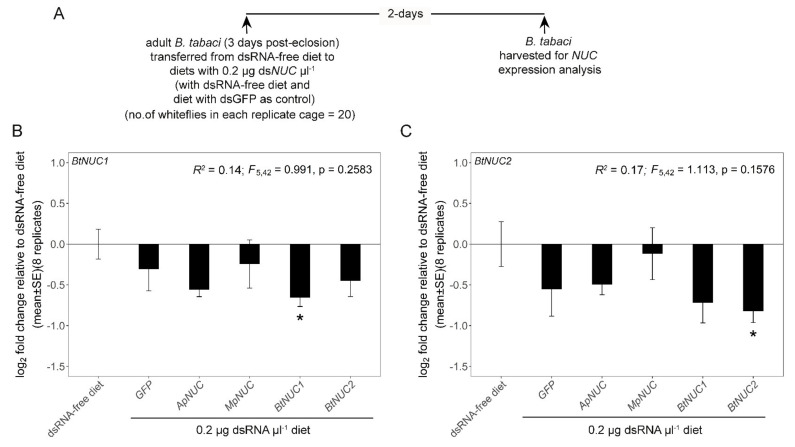
Activity of aphid ds*NUC* against expression of *B. tabaci NUC* genes. (**A**) Experimental design. (**B**) *B. tabaci NUC1* gene expression and (**C**) *B. tabaci NUC2* gene expression. Pooled data for two iterations, each iteration comprising four replicate cages for each treatment, are shown. Linear model statistics were employed using dsRNA-free diet as the intercept to identify significant effects of various dietary dsRNAs on *NUC* expression. Asterisks identify treatments that are significantly different from ds*GFP*-free diet at α = 0.05. *ApNUC*, *A. pisum NUC*; *BtNUC1*, *B*. *tabaci NUC1*; *BtNUC2*, *B*. *tabaci NUC2*; *MpNUC*, *M. persicae NUC*.

The final experiments investigated the effect of heterologous ds*NUC*s on *NUC* expression. For the analysis of the two aphid species, ds*ApNUC* and ds*MpNUC* at 0.2 µg µL^−1^ was administered to 5-day-old aphids of *A. pisum* clone SC_37 and *M. persicae*, with dsRNA-free diet and ds*GFP* as negative controls (Figure 3A). Five replicate cages, each containing 5 aphids, were used. The analysis of *dsNUC* cross-reactivity was then extended to the mealybug *P*. *maritimus* (ca. 30 days old) and the whitefly *B*. *tabaci* (3 -days post-eclosion) (Figure 4A and Figure 5A). The homologous *P. maritimus dsNUC* (NCBI accession number MT187988.1, referred to as *NUC1* in [39]), the two homologous *B. tabaci NUC1* and *NUC2* (NCBI accession numbers KX390872.1 and KX390873.1, respectively [37]) and the heterologous ds*NUC* from both aphids, were administered at 0.2 µg µL^−1^ over 2 days. The experiments included two negative controls: dsRNA-free diet and ds*GFP*. Twenty insects were administered to each diet cage, and the harvested insects were stored at −80 °C prior to analysis. Due to limited supply of insects, the experiments comprised two iterations: three replicates for the first iteration and two replicates for the second iteration for *P. maritimus*; and four replicates in each of the iterations for *B. tabaci*. The data for the two iterations for each insect species were pooled, following confirmation that the data did not differ significantly between two iterations (*p* = 0.26 for *P*. *maritimus*; *p* = 0.35 and 0.69 for *B*. *tabaci NUC1* and *NUC2*, respectively).

### 2.7. Statistical Analysis

R software version 3.5.1 was used to analyze the data (R Core team). For the analysis of the response of *A. pisum* to dietary ds*GFP*, variation in aphid weight was analyzed by non-parametric Kruskal–Wallis test (following demonstration of heterogeneity of variances), and non-parametric Dunn’s test for treatment comparisons to the dsRNA-free control using PMCMRplus package version 1.4.4 [47]. All other datasets were normally distributed with homogeneous variances. Honeydew production by pea aphids was investigated as a linear model [48] followed by Dunnett’s post hoc test using emmeans package version 1.3.3 [49]. The log2-fold variation in gene expression was analyzed as a linear model using the lm function in lme4 package version 1.1–2.1 [48] with treatments as categorical predictors.

## 3. Results

### 3.1. Optimization of the Dietary Concentration of dsRNA

To identify the optimal concentration of dietary dsRNA for analysis of the reactivity of dsRNA in different insect species, we first investigated the sequence-non-specific effects of dsRNA at different concentrations. The experiments were conducted on *A. pisum* clone CWR09/18, which has been used extensively in previous feeding analyses and RNAi studies in our laboratory [40,43,44]. The insects were fed for two days on diets containing 0–1.0 μg ds*GFP* μL^−1^ (with no sequence homology to insect genomes), and on water as a control. Compared to aphids on the ds*GFP*-free diet, the final weight of the aphids was significantly depressed on the water-only control but not on the diets containing ds*GFP* (Figure 1B). However, the weight of honeydew produced by the insects, which provides a useful index of the amount of food consumed by aphids [50], was significantly depressed while they were on diets containing 0.5 and 1.0 μg ds*GFP* μL^−1^, but not on diets with lower ds*GFP* concentrations, relative to the ds*GFP*-free diet (Figure 1C). The aphids on the water control diet produced negligible amounts of honeydew, reflecting the absence of phagostimulatory sugar. These data suggest that ds*GFP* at ≥0.5 μg μL^−1^ has an antifeedant effect on the insects, and that concentrations <0.5 μg μL^−1^ should be used to avoid sequence-non-specific effects of dsRNA.

We then investigated the expression knockdown in aphids administered dsRNA against the nuclease gene *NUC* at concentrations in the range 0.05–0.4 µg ds*NUC* µL^−1^. The experiments were conducted on *A*. *pisum* clone SC_37 and *M*. *persicae* clone GPA, which are highly susceptible to RNAi [38,44]. For both species, *NUC* expression was significantly reduced, on average by ca. 50% relative to the dsRNA-free diet, on diets containing 0.2 µg µL^−1^ but no other dsRNA concentration tested (Figure 2B,C). Subsequent experiments investigating the efficacy of aphid ds*NUC* against other insect species focused on dsRNA at 0.2 µg µL^−1^. Possible reasons for the low efficacy of dsRNA at 0.4 µg µL^−1^ are considered in the Discussion.

### 3.2. dsNUC Cross-Reactivity

We first compared the gene expression of *NUC* in *A. pisum* SC_37 and *M. persicae* GPA administered ds*ApNUC* or ds*MpNUC* at 0.2 µg µL^−1^. These genes have high sequence identity, and 77 of the 308 predicted 21 nt siRNA sequences derived from the two *dsNUC*s are perfect matches (Table 1, Figure S1). For both species, the homologous ds*NUC* significantly reduced *NUC* expression relative to dsRNA-free control, but the heterologous ds*NUC* had no significant effect (Figure 3B,C).

We then extended the analysis to investigate the expression of *NUC* genes in the mealybug *P*. *maritimus* and the whitefly *B*. *tabaci* administered heterologous ds*NUC* from the two aphid species. The ds*NUC*s of these species have low sequence identity with aphid *dsNUCs* and share no perfectly matching 21-mer sequences (Table 1; Figures S2–S7). We predicted that in the absence of sequence-non-specific effects, the aphid ds*NUC*s would not significantly affect the expression of *NUC* genes in these insects, while the homologous ds*NUC* would mediate *NUC* expression knockdown. The results for *P*. *maritimus* were fully consistent with this expectation (Figure 4B). Our analysis of *B*. *tabaci* took into account that two nuclease genes, *BtNUC1* and *BtNUC2*, are expressed in the gut [40]), unlike the other species with a single gut-expressed *NUC* gene. *BtNUC1* and *BtNUC2* have low sequence identity and share no 21-mer sequences (Table 1 and Figure S8). As predicted, the expression of both *B. tabaci NUC* genes was significantly reduced only in whiteflies administered the homologous ds*NUC* (Figure 5B,C). However, a nonsignificant trend of reduced *NUC* expression was evident in several treatments, with mean transcript abundance being more than halved for *BtNUC1* in insects administered ds*ApNUC*, and for *BtNUC2* in whiteflies administered ds*GFP* and ds*BtNUC1.* These findings raise the possibility of sequence-non-specific effects of dsRNA administered at relatively low concentrations in *B. tabaci*.

## 4. Discussion

The successful application of RNAi for pest control is a balancing act, requiring high efficacy against pest species with minimal deleterious effects on non-target organisms. Achieving this balance requires a firm understanding of both sequence-dependent and sequence-non-specific effects of dsRNA, while recognizing that responses to RNAi triggers can vary widely between different species. This variation is particularly evident for insects, given substantial reported differences in RNAi susceptibility between insect orders [3,4,5], related insect species [11,51] and even within species [44,52,53]. This study on the specificity of gene expression knockdown in plant sap-feeding insects has revealed both sequence-non-specific effects at high concentrations of administered dsRNA (≥0.5 μg μL^−1^ diet) and sequence-dependent effects on gene expression at a lower concentration (0.2 μg μL^−1^). In this Discussion, we address the implications of these findings, particularly in relation to the application of RNAi for the control of plant sap-feeding crop pests.

Insight into sequence-non-specific effects of dsRNA was obtained from the analysis of pea aphids *A. pisum* administered ds*GFP* at different dietary concentrations (Figure 1). The significantly reduced food consumption from diets containing ≥0.5 μg ds*GFP* μL^−1^ diet, without significant concomitant reduction in weight or any mortality over two days, is strongly indicative of an antifeedant effect of the dsRNA. Factors contributing to reduced feeding may include high viscosity of the liquid diet [54] arising from the addition of dsRNA at high final concentrations, and possible residual chemical contaminants in the administered dsRNA (despite our use of rigorous protocols for dsRNA purification, see methods). Plant sap-feeding insects may be more susceptible to these effects than chewing insect pests, such as Lepidoptera and Coleoptera. This is because dsRNA can be added to solid foods with minimal effects on the physical properties of the diet, and plant sap-feeders are notoriously intolerant of low concentrations of impurities in artificial diets [55]. Nevertheless, our findings raise the possibility that antifeedant effects may contribute to some of the observed sequence-non-specific effects of dsRNA on other insects, including increased mortality and global changes in gene expression [11,23]. If further research demonstrates that chemical contaminants contribute to the sequence-non-specific effects of dsRNA preparations, this issue should be addressed in the design of commercial preparations using exogenously-applied dsRNA for insect pest control [56,57,58].

An alternative and widely-adopted approach to investigate sequence-non-specific effects of dsRNA is to include ds*GFP* (or dsRNA against another sequence with no homology to the insect genome) in experiments quantifying dsRNA effects on expression of a target gene [3,5]. For example, the non-significant effects of ds*GFP* on *NUC* expression by the insects tested in this study (Figure 3, Figure 4 and Figure 5) offers a first indication that, at 0.2 μg μL^−1^, dsRNA does not induce substantial sequence-non-specific effects on expression of this test gene in these insects. In support of this interpretation, the mortality of all the insect species studied here was negligible in these experiments, although other indices of insect performance, including developmental rate and fecundity, were not investigated. Nevertheless, as highlighted in the Results section, the expression of the *B. tabaci* nuclease genes *NUC1* and *NUC2* was reduced two-fold by dsRNAs with no predicted sequence-dependent cross-reactivity, including ds*GFP* for *BtNUC2*. Although these differences were not statistically significant, they raise the possibility that *B. tabaci* and other whiteflies may be particularly susceptible to sequence-non-specific effects of dsRNA. These considerations indicate the importance of further research to quantify the patterns and underlying processes of non-sequence dependent effects of dsRNA in different insects, and also to ensure that RNAi studies have the appropriate controls to discriminate between sequence-dependent and sequence-non-specific effects.

An issue arising from these considerations is our finding that expression knockdown of the *NUC* gene in both aphid species was lower in aphids administered the homologous ds*NUC* at 0.4 μg μL^−1^ diet, relative to the lower concentration of 0.2 μg μL^−1^. Antifeedant effects of dsRNA at 0.4 μg μL^−1^ may have contributed to this effect. Additionally or alternatively, the regulatory circuits controlling *NUC* gene expression may be complex, with a more robust or more rapid compensatory upregulation of expression triggered by ds*NUC* at 0.4 than at 0.2 μg μL^−1^. Further experiments investigating the time course of *NUC* expression and food consumption in aphids administered different ds*NUC* concentrations should resolve these issues. More generally, these results demonstrate that the efficacy of RNAi is not necessarily enhanced by increasing the dose of dsRNA, and reinforce the importance of determining the optimal concentration for each target gene [59].

The sequence-dependent effects of dsRNA are also critical for the effective and safe application of RNAi in the control of plant sap-feeding insect pests. As considered in the Introduction, the number and length of exactly matching siRNA sequences derived from a heterologous dsRNA that are required for successful expression knockdown can vary with the target gene and insect species. Although the cross-reactivity of dsRNAs in plant sap-feeding insects has not been investigated extensively, evidence that heterologous dsRNA can confer effective RNAi is provided by a study of *CP19*, an essential gene coding aphid cuticle protein 19 which protects the insects against cuticular water loss and desiccation [60]. Across the three aphid species studied, *CP19* had 94% sequence identity (the number of matching 21-mer sequences was not reported) and ds*CP19* matching the *CP19* sequence of *Aphis citricidus* reduced *CP19* expression in both *A. pisum* and *M. persicae*, with attendant mortality effects. The different result in our study, where no detectable cross-reactivity in expression of *NUC* genes of *A. pisum* and *M. persicae* was obtained, may reflect a critical difference in the number of perfectly matching siRNA molecules (just 25% of the siRNAs met this criterion, despite 92% sequence identity of the ds*NUC*s constructed for the two species; see Table 1). Other factors may also be important, including choice of gene, aphid genotype, culture conditions and possibly dsRNA concentration. In [60], ds*CP19* was administered at 1.5 μg μL^−1^ by petiole dip, raising the possibility of sequence-non-specific effects, although the concentration ingested by the aphids was not determined. More broadly, the interesting differences between the findings of [60] and this study illustrate how further investigation are required on the species-specificity of dsRNA against plant sap-feeding insect pests.

We conclude with two points. First, developing an understanding of the factors that promote sequence-non-specific effects of dsRNA will be crucial to enable accurate predictions of the deleterious effects of heterologous dsRNAs on non-target, plant sap-feeding insect species. This study adds to the conclusions from prior research on various insects that these effects tend to be mediated by high dsRNA concentrations [3,5,25], and it also demonstrates that dsRNA formulations can have significant antifeedant effects for orally-delivered RNAi. Second, there is a need to extend research on the relationship between the number of matching siRNA sequences and RNAi efficacy of dsRNA molecules [16,61,62], so that effective dsRNAs can be designed against single or closely-related pest species that vary in sequence identity with the target gene. Attention to these issues will facilitate the development and deployment of RNAi-based solutions for the management of plant sap-feeding and other insect pests.

## Figures and Tables

**Table 1:** Sequence identity of *NUC* gene sequences and ds*NUC* sequences and predicted cross-reactivity of ds*NUCs.*

Insect Species	% Sequence Identity of *NUC* Gene Sequences/ds*NUC* Sequences				d*sNUC* Length (nt)	Predicted Cross Reactivity of ds*NUC* Number of Perfect Matching 21 nt siRNA Molecules (% of Total siRNA Molecules) ^1^			
	*Ap*	*Mp*	*Pm*	*Bt* (*NUC1*)		*Ap*	*Mp*	*Pm*	*Bt* (*NUC1*)
*Ap*					328				
*Mp*	88/92				328	77 (25%)			
*Pm*	39/35	39/38			250	0	0		
*Bt* (*NUC1*)	43/47	43/47	31/41		402	0	0	0	
*Bt* (*NUC2*)	46/40	45/40	43/34	38/40	400	0	0	0	0

^1^ Calculated using siRNA-Finder (siFi) software [14].

## Data Availability

All data generated or analyzed during this study are available from the corresponding author on reasonable request.

## Supplementary Materials

The following are available online at https://www.mdpi.com/2073-4425/12/3/407/s1, Table S1. Primer sequences. Figure S1. Alignment of *A. pisum* *NUC* (NCBI accession number: XM_003242604.4) and *M. persicae* *NUC* (NCBI accession number: XM_022327343.1). Red font represents the ds*NUC* sequence and the yellow background represents perfect matches greater than or equal to 21 nt between *A. pisum* ds*NUC* and *M. persicae* ds*NUC*. Figure S2. Alignment of *A. pisum* *NUC* and *P. maritimus* *NUC* (NCBI accession number: MT187988.1). Red font represents the *A. pisum* ds*NUC* sequence. Figure S3. Alignment of *A. pisum* *NUC* and *B. tabaci* *NUC*1 (NCBI accession number: KX390872.1). Red font represents the *A. pisum* ds*NUC* sequence. Figure S4. Alignment of *A. pisum* *NUC* and *B. tabaci* *NUC*2 (NCBI accession number: KX390873.1). Red font represents the *A. pisum* ds*NUC* sequence. Figure S5. Alignment of *M. persicae* *NUC* and *P. maritimus* *NUC*. Red font represents the *M. persicae* ds*NUC* sequence. Figure S6. Alignment of *M. persicae* *NUC* and *B. tabaci* *NUC*1. Red font represents the *M. persicae* ds*NUC* sequence. Figure S7. Alignment of *M. persicae* *NUC* and *B. tabaci* *NUC*2. Red font represents the *M. persicae* ds*NUC* sequence. Figure S8. Alignment of *B. tabaci* *NUC*1 and *B. tabaci* *NUC*2. Red and green represent *B. tabaci* ds*NUC*1 and ds*NUC*2 sequences.
