# Supplementary material for: Non-Target Effects of dsRNA Molecules in Hemipteran Insects
Source: Genes (Basel). 2021 Mar 12;12(3):407. doi: 10.3390/genes12030407 (PMC8000911; doi:10.3390/genes12030407)
Supplement: Supplementary file 1 [file genes-12-00407-s001.zip › Supplementary_information/Supp_figures_013021b.pdf]

```
A. pisum-NUC        ------ATGCATTCAACTGCATTAATTACTGCAGTATGTTTAGTCATTTGTTCGTGTCTT
M. persicae-NUC     ATGTATTCAATGTATTCAATTGCATTCACAGCAGTATGTTTAATCATTTGTTCGTGTCTG
                                *   *      * * ** ************ **************** 

A. pisum-NUC        CTTCCGACCATCAATGCCAAGAAAATCAATTATCAAAATGTTAAAAACGAGGACGACTGT
M. persicae-NUC     CGTCCGACCATTTATGCCAAGGAAATCAAT---CGACCGGTTAAAAACGACGACGACTGT
                    * *********  ******** ********   * *   *********** *********

A. pisum-NUC        TCGTTATGGGTGGCCGAAAAAGACGAGCCCAAAATGCCCATACCGTTCACGAGCAACGGC
M. persicae-NUC     TCGCTATGGGTGGCCGAAAAAAACCAGTCCAAAATGCCCATACCGTTCACGAGCAACGGC
                    *** ***************** ** ** ********************************

A. pisum-NUC        AAGCGGTACGCGATCATCTATCCGAACGCCAAGGGCCGGCTGGACGTGAAGAAAGGGCGT
M. persicae-NUC     AAACAGTACGCGATCATCTATCCGGATGAAAAGGGCAGGCTGCACGCGGAGAAAGGGCGC
                    ** * ******************* * *  ****** ***** *** * ********** 

A. pisum-NUC        AGTTTCAAGCTGTCGTGCGCCACAGCCAAGTTCGCGTCGAGCGAGCTGCAACGGAACGGC
M. persicae-NUC     AGTTTCAAGCTGTCGTGCGCCACTGCCAAGTTCGCGTCGAGCGAACTGCGGCGGGACGAT
                    *********************** ******************** ****  *** ***  

A. pisum-NUC        ACCTCCGAAGTGTTGGTCACGTGCGTCGGCATCGACGTCCTCGCGTACCGGGGCCAGACG
M. persicae-NUC     TCCTCCGACGCGTTGGTCACGTGCGTCGGCGGCGACGTGCTCGCGTACCGAGGCCAGACG
                     ******* * *******************  ****** *********** *********

A. pisum-NUC        TACCGGTACGAAGACTTCCAGTGCGACGGCATGCCCAAGTCGGAGCTCCGAGTCACCGAC
M. persicae-NUC     TACCGGTACGCCGCCTTCCAGTGCGACGGCATGCCCAAGTCGGAGCTCCGCGTCACCGAC
                    **********  * ************************************ *********

A. pisum-NUC        GATCGGTGCCAGCCGGCCAACTACACCGTGGCCGTGGTCGGTTTCCAGACGGACCACGCA
M. persicae-NUC     GAAGTGTGCCAGCCGGCCAACTACACCGTGGCCGTGGTCGGTTTCCGGACGGATCGCGCG
                    **   ***************************************** ****** * *** 

A. pisum-NUC        TTCCTCCGGCTGTACGGCATGTGTTTCGACAAGTCGACCAAGAACAGCCTGTACACTTGG
M. persicae-NUC     TTCCTCCGGCTGTACGGCATGTGCTTCGACAAGTCGACCAAGAACAGCCTGTACACTTGG
                    *********************** ************************************

A. pisum-NUC        TACGACGCCCGGGCGCCGTACTACGACAACCACCAGAAGTACAGCAAGCGACCGGCGTTC
M. persicae-NUC     TACGACGCCCGGTCGCCGTACTACGACAACCACCAGAAGTATAGCAAGCGACCGTCGTTC
                    ************ **************************** ************ *****

A. pisum-NUC        ATTAAGTCCAAAGAGCTGTACGGCAACACGGACGTGAACAGGAAATACACTTTCAAAGAA
M. persicae-NUC     AACAAAACCAAAGAGCTGTACGGCAACACGGACGTGAACAAGAAATACACTTTCAAAGAA
                    *  **  ********************************* *******************

A. pisum-NUC        CAGAAAAAAACGGTGGCGAAAATACTCAGATCGGACGAACTTGCGGATAAGTACATAAGG
M. persicae-NUC     CAGAGAAACACGGTGGCGACAATACTCAGATCGGACGAACTTGCGGATAAGTACATAAGG
                    **** *** ********** ****************************************

A. pisum-NUC        AATGATAACCAACACTCGCTGTCCCGGGGCCATTATGCAGCCAAAGCTGACTTCTTCTTT
M. persicae-NUC     AATGATAATAAACACTTCCTGTCCCGGGGCCATTACGCAGCCAAAGTTGACTTTTTCTTT
                    ********  ******  ***************** ********** ****** ******

A. pisum-NUC        GATTTCGAACAGATTTCAACGTTTTACTATGCGAACGTAGCCCCACAGTGGCAGATATTC
M. persicae-NUC     GCTTATGAACAAACAGCAACGTTTTACTATGCGAACGTTGCCCCGCAGTGGCAGATCTTC
                    * **  ***** *   ********************** ***** *********** ***

A. pisum-NUC        AACGGTGACATGTGGGCCGATCTGGAGCAGTCAACCCGGTCAAAGTTAAGCAAAGGAAAC
M. persicae-NUC     AACGGTGACATGTGGGCCGATCTGGAATCGACTACCCGGTCAAAGTTGAGCAAGGAAAAC
                    **************************   * * ************** ***** * ****

A. pisum-NUC        GGCACGTCCACACATGTGATCGTCACCGGTACGTACGACACATGCACACTGGCCGACGTG
M. persicae-NUC     GGCA---CCAGACACGTGATCGTCACCGGCACGTACGAAGTGTGCACGCTGCCCGACGTG
                    ****   *** *** ************** ********    ***** *** ********

A. pisum-NUC        GACAACGTCCAACAACCACTGTACCTTGACCTGCCTGGTGCCATACGCGTGCCGCTGTTC
M. persicae-NUC     GACGACATCCAACAACCACTGTACCTTGACCTGCCCCGTAGCATACCCGTGCCGCTGCTC
                    *** ** ****************************  **  ***** ********** **

A. pisum-NUC        TACTGGAAGCTGCACTACGATGTGGACGCGGCGGACGGCATCGTGTACATCGGCCTCAAC
M. persicae-NUC     TACTGGAAGCTGTACTACGATGTGGACACGGAGGACGGCATCGTGTATATCGGTCTCAAC
                    ************ ************** *** *************** ***** ******
```

```
A. pisum-NUC         AACCCGTACAAGGAGATCGACGACAGCGTGTACATATGTCCTAACATATGCCCGGACGGT
M. persicae-NUC      AACCCCTACAAGGAGATCGACGACAGCGTGTACATATGTCCTAACACATGTCCGGACGGT
                     ***** **************************************** *** *********

A. pisum-NUC         TACCATGGTCGCGGTTACCAGGGCCGCGATCACCGAAACAATAAGGCAGAGACCGGACCC
M. persicae-NUC      TACCATGGTCGCGGTTACCAGGGCCGCGATCCCCAAAATCATCCATCAGAGACCGAACCA
                     ******************************* ** ***  **    ********* ***

A. pisum-NUC         GGCCGCGATCACCGGAACGACCCCGAACCGGACGCCAACGACGGACTCATCTACTGCTGT
M. persicae-NUC      GACCGCGGTCACCGGAACGACCCCGAACCGGACGCCAACGACGGGCTCATCTACTGCTGT
                     * ***** ************************************ ***************

A. pisum-NUC         ACCAAAAAGTCGTTCGAAGAGGTTTACGGTGAGCTGGACCCAATCGTGTACAGACAGCTG
M. persicae-NUC      ACCAAAGAGTCATTCGAAGATGCTTACGGCAAGCTGGACCCAATCGTATACCGACAGCTC
                     ****** **** ******** * ******  **************** *** *******

A. pisum-NUC         ATG---
M. persicae-NUC      ATGTGA
                     ***
```

Supplementary Figure S1. Alignment of *A. pisum NUC* (Accession number: ACYPI008471) and *M. persicae NUC* (Accession number: MYZPE13164). Red fonts represent ds*NUC* sequence and yellow background represents perfect matches greater than or equal to 21 nt between *A. pisum* ds*NUC* and *M. persicae* ds*NUC*.

```
A. pisum-NUC            ATGCATTCAACTGCATTAATTACTGCAGTATGTTTAGTCATTTGTTCGTGTCTTCTTCCG
P. maritimus-NUC        -----------------------------------------------GTTTTCTCATTAA
                                                                       ** *  ** *

A. pisum-NUC            ACCATCAATGCCAAGAAAATCAATTATCAAAATGTTAAAAACGAGGACGACTGTTCGTTA
P. maritimus-NUC        ACGATCATG------------------------------TACCAAAAACCATCATCTTGA
                        ** ****                                 ** *  *    *  ** * *

A. pisum-NUC            TGGGTGGCCGAAAAAGACGAGCCCAAAATGCCCATACCGTTCACGAGCAACGGCAAGCGG
P. maritimus-NUC        TGAGTGCAA------------CCCAAAATAAC----------------------------
                        ** ***               ********  *

A. pisum-NUC            TACGCGATCATCTATCCGAACGCCAAGGGCCGGCTGGACGTGAAGAAAGGGCGTAGTTTC
P. maritimus-NUC        ---CAAATGCTCTACCCGAGCGAATCCAATACAATCAGAATGCGTACCGGGACCCGGTTC
                              **  **** **** **            *     **   *  ***    * ***

A. pisum-NUC            AAGCTGTCGTGCGCCACAGCCAAGTTCGCGTCGAGCGAGCTGCAACGGAACGGCACCTCC
P. maritimus-NUC        AAGGTAAGCTGCGGCGATAAAGACTTCAAGAAAAAATTCAAAAAATCACCAAGAACCAAA
                        *** *    **** *       * ***  *   *         **       * ***

A. pisum-NUC            GAAGTGTTGGTCACGTGCGTCGGCATCGACGTCCTCGCGTACCGGGGCCAGACGTACCGG
P. maritimus-NUC        GAAGTCCAAGCACGTTGCAATTCCAAAGACATAATCAATGTCGAAGGCGAACGAATACGT
                        *****    *     ***     **  *** *  **     *   *** *       **

A. pisum-NUC            TACGAAGACTTCCAGTGCGACGGCATGCCCAAGTCGGAGCTCCGAGTCACCGACGATCGG
P. maritimus-NUC        TTTCGAGAACTAGAATGTCAAAGTTTTCCAACATCCAAACCCCAAAAAAGAGAAAATAAA
                        *    ***  *  * **  *  *  * ** *  **  * * ** *   *  **  **

A. pisum-NUC            TGCCAGCCGGCCAACTACACCGTGGCCGTGGTCGGTTTCCAGACGGACCACGCATTCCTC
P. maritimus-NUC        AAATGCCATGGAAACAACACGCTCTTCGATATAGGATTTCCAACCCGCGACAATTTTTTG
                              *  *  *** ****  *   **   * ** ** *  **   * **   **  *

A. pisum-NUC            CGGCTGTACGGCATGTGTTTCGACAAGTCGACCAAGAACAGCCTGTACACTTGGTACGAC
P. maritimus-NUC        GATATGATTCGAGTATGTTTCGATGAACTACAACAGGAGTCTCGTTACACCTGGTATGAT
                            **    *  * ********  *        ** *    *  ***** ***** **

A. pisum-NUC            GCCCGGGCGCCGTACTACGACAACCACCAGAAGTACAGCAAGCGACCGGCGTTCATTAAG
P. maritimus-NUC        TCGTCGATGCTACCAACAGGACAT---CAGAGTAACGTAGGAAGACCTCGATTCGTCC--
                         *   *  **        *   *    ****   **       ****    *** *

A. pisum-NUC            TCCAAAGAGCTGTACGGCAACACGGACGTGAACAGGAAATACACTTTCAAAGAACAGAAA
P. maritimus-NUC        -ACGATAATCTCTACAGGTTCCCGGTCG---ACGAGGTGTACAAGAGTAGTTACCAGCAC
                          * *  * ** *** *   * *** **   **  *   ****     *   * *** *

A. pisum-NUC            AAAACGGTGGCGAAAATACTCAGATCGGACGAACTTGCGGATAAGTACATAAGGAATGAT
P. maritimus-NUC        GATTGGTTTACCAAATTGTTAAAATCTCGAGAAAAAGCTGATCAATATATCAAAAACGAC
                         *   * *  * *** *  * * ***    ***   ** *** * ** ** *  ** **

A. pisum-NUC            AACCAACACTCGCTGTCCCGGGGCCATTATGCAGCCAAAGCTGACTTCTTCTTTGATTTC
P. maritimus-NUC        GGCGAACATTTTCTGTCCAGAGGGCATCTTACGCCAAAGGCTGATATGGTTTATGGATCG
                          * **** *  ****** * ** ***  * *  * ** *****  *  * * **  *

A. pisum-NUC            GAACAGATTTCAACGTTTTACTATGCGAACGTAGCCCCACAGTGGCAGATATTCAACGGT
P. maritimus-NUC        GAACAATCAGCAACTTTCCATTATATCAACGTGGCACCTCAGTGGCAAGGTTTCAACGGT
                        *****     **** **  * ***   ***** ** ** ********    *********

A. pisum-NUC            GACATGTGGGCCGATCTGGAGCAGTCAACCCGGTCAAAGTTAAGCAAAGGAAACGGCACG
P. maritimus-NUC        GGTAATTGGAATAAAGTTGAACAAAGTGCAAGAGAAGAGCTCGAGAAAAAGGATAA----
                        *  *  ***    *  * ** **     *  *   * ** *    ***    *

A. pisum-NUC            TCCACACATGTGATCGTCACCGGTACGTACGACACATGCACACTGGCCGACGTGGACAAC
P. maritimus-NUC        --AAGATACCGTGTGGTAACCGGAACCTACGGAGTGGCCACGCTACCAGACGTAAACAAC
                           * * *     * ** ***** ** ****       *** **  * *****  *****

A. pisum-NUC            GTCCAACAACCACTGTACCTTGACCTGCCTGGTGCCATACGCGTGCCGCTGTTCTACTGG
P. maritimus-NUC        AACGAACAAGAACTCTACCTCTACGAAGACGAGAACAAAAACCCATTGCTCA--------
                          * *****  *** *****  **      *    ** *  *     ***

A. pisum-NUC            AAGCTGCACTACGATGTGGACGCGGCGGACGGCATCGTGTACATCGGCCTCAACAACCCG
P. maritimus-NUC        ------------------------------------------------------------
```

```
A. pisum-NUC          TACAAGGAGATCGACGACAGCGTGTACATATGTCCTAACATATGCCCGGACGGTTACCAT
P. maritimus-NUC      ------------------------------------------------------------

A. pisum-NUC          GGTCGCGGTTACCAGGGCCGCGATCACCGAAACAATAAGGCAGAGACCGGACCCGGCCGC
P. maritimus-NUC      ------------------------------------------------------------

A. pisum-NUC          GATCACCGGAACGACCCCGAACCGGACGCCAACGACGGACTCATCTACTGCTGTACCAAA
P. maritimus-NUC      ------------------------------------------------------------

A. pisum-NUC          AAGTCGTTCGAAGAGGTTTACGGTGAGCTGGACCCAATCGTGTACAGACAGCTGATG
P. maritimus-NUC      ---------------------------------------------------------
```

Supplementary Figure S2. Alignment of *A. pisum NUC* and *P. maritimus NUC* (Accession number: MT187988.1). Red fonts represent *A. pisum* ds*NUC* sequence.

```
A. pisum-NUC        ------------------------------------------------------------
B. tabaci-NUC1      GCTGATGAAACCGGAAATGAGGTCCGTGCAGTCAGAGTGGAAGTAGAAGAGGATGATGAG

A. pisum-NUC        ------------------------------------------------------------
B. tabaci-NUC1      AAAACGGCAGAAGTGAAGATGGAAATGGGAATGGGTGAAACCGGAGACGGTTTCCGTACC

A. pisum-NUC        ------------------------------------------------------------
B. tabaci-NUC1      ATGAAGCCCGATCATTTCAGTGGTAAACTCAAAGTTGGTAAGCAGGGAGGAGCCGCGTCG

A. pisum-NUC        ------------------------------------------------------------
B. tabaci-NUC1      ACAACTACGAATGGAGGAATTAAGAATGATTTTGAGCTGAGAAACTTGAAAGTCGTGCCC

A. pisum-NUC        ------------------------------------------------------------
B. tabaci-NUC1      GAAAAAGAACCGACGGATACGGAAGTTGACACGTCCGTTACCGTAAAGGAGAGCGGAGGT

A. pisum-NUC        --------------------------------ATGCATTCAACTGCATTAATTACTGCAG
B. tabaci-NUC1      GACTCGAAGCTCCTAAGTTTGTTCGCCGGTTATCGGATGCGAATACAAGAAGGAGTCCAC
                                                      * ** * * * **  **  * * **

A. pisum-NUC        TATGTTTAGTCATTTGTTCGT-GTCTTCTTCCGACCATCAATGCCAAGAAAATCAATTAT
B. tabaci-NUC1      AATGTTGCCTTCGACTTGCCTTTAAATTTTAGGAGCACTGGTAGCAGGAAACTCGCTCCT
                     *****   *      * * *     * **  ** **    *  ** **** **  *  *

A. pisum-NUC        CAAAATGTTAAAAACGAGGACGACTGTTCGTTATGGGTGGCCGAAAAAGACGAGCCCAAA
B. tabaci-NUC1      CTTGTAGTTGAAAATGTCCGCGCGTTGGGCACATTCACAACCGGAAACAACAT-----GA
                    *     *** **** *    **  *       **      *** ***  **        *

A. pisum-NUC        ATGCCCATACCGTTCACGAGCAACGGCAAGCGGTACGCGATCATCTATCCGAACGCCAAG
B. tabaci-NUC1      GTGGTACAAACGGTCGCGTGACGCGTGA--------TGCATCAAACATTAGAATAAGACA
                     **     * ** ** ** *   **  *           ****   **  ***    *

A. pisum-NUC        GGCCGGCTGGACGTGAAGAAAGGGCGTAGTTT-------------CAA----------GC
B. tabaci-NUC1      GGAAGAGCAGACACCAATCAGAGACGGAATGAAAATGATACTAATGAAAAAAATGTTTGC
                    **  *    ***   **  *  * ** * *                **          **

A. pisum-NUC        TGTCGTGCGCCACAGCCAAGTTCG-------------------------CGTCGAGCGAG
B. tabaci-NUC1      AACCGGAAGCGACGCCAGCCTTCTCGATGACGGTTACAGCTTAAAGCCGCATGATGCACG
                       **   ** **  *    ***                          * *   **  *

A. pisum-NUC        CTGCAACGGAACGGCACCTCCG--------AAGTGTTGGTCACGTGCGTCGGCATCGACG
B. tabaci-NUC1      CTTCACCGGAAACGATGTCAGGCTGACTGAAAGCAGTGCTAGCCTCACTGCGAGTAGCAA
                    ** ** *****  *       *        ***   ** *  * *   *  *  * *

A. pisum-NUC        TCCTCGCGTACCGGGGCCAGACGTACCGGTACGAAGACTTCCAGTGCGACGGCATGCCCA
B. tabaci-NUC1      -----------CATCCGCATCGGTGGAATTACGAACATTAACGGGAACGACGGCGGC-TT
                               *     **   **     ****** * *  * *       *   **

A. pisum-NUC        AGTCGGAGCTCCGAGTCACCGACGATCGGTGCCAGCCGGCCAACTACACCGTGGCCG-TG
B. tabaci-NUC1      CATCGGAGGA-AGTGTAACCCC---TGCCGAAAATGCAGCTAGCTCCGCCGGAAATAGTG
                      ******    * ** ***     *       *  * ** * ** * ***       **

A. pisum-NUC        GTCGGTTTCCAG-ACGGACCACGCATTCCTCCGGCTGTACGGCATGTGTTTCGACAAGTC
B. tabaci-NUC1      TAAGCTTTACCGGAAATATTGAAGGTCTAATCGAAAGTACCACCATTCACACTGAAGGTG
                       * *** * * *   *       *     **   ****  *   *    *   * **

A. pisum-NUC        GACCAAGAACAGCCTGTACACTTGGTACGAC---------GCCCGGGCGCCGTACTACGA
B. tabaci-NUC1      AACCCAGCTATACCTATACTGGTTCGAACCTTTCCGGAGGTGACGCCAGCTTTACTAGAA
                     *** **     *** ***   *   *                **   **  *****  *

A. pisum-NUC        CAACCACCAGAAGTACAGCAAGCGACCGGCGTTCATTAAGTCCAAAGAGCTGTACGGCAA
B. tabaci-NUC1      ATGGCGCCGGCTCCACCGGAAATGGCC-----TCAGCCTTTCCG----AATATAGTGCTA
                        * ** *    ** * **  * **     ***     ***       * **  ** *

A. pisum-NUC        CACGGACGTGAACAGGAAATACACTTTCAA-AGAACAGAAAAAAACGGTGGCGAAAATAC
B. tabaci-NUC1      AT--TCCATCAGGAGCTATACCGCTTTCGCTCGGAACGGCCACAACTCTGCTGGAAATGC
                          * * *  **  *   * *****    * *  *   * ***  **  * **** *
```

```
A. pisum-NUC          TCAGATCGGACGAA--------------------CTTGCGGATAAGTACATAAGGAATGA
B. tabaci-NUC1        CCATGACCCGAGTCTCACCGGAAATGACCCCAGTCTTACCGGAAATGACCCCAGTCTTAC
                       **   *    *                      *** * *  **  **   **   *

A. pisum-NUC          TAACCA-ACACTCGCTGTCCCGGGGCCATTATGCAGCCAAAGCTGACTTCTTCTTTGATT
B. tabaci-NUC1        CGGAAATGACCCCAGTCTTACCGGAAATGACCTCAGTCGTACCGGAAATGGCCCTAGTCT
                           *    * *  * *  * **         *** *  * * **  *   * * *  *

A. pisum-NUC          TCGAACAGATTTCAACGTTTTACTATGCGAACGTAGCCCCACAGTGGCAGATATTCAACG
B. tabaci-NUC1        TTCCAGAGATGACTCCAATTTTCCCAGAAATAGT------------GCCAGTCTTCCAAG
                      *   * ****  *  *  *** *   *  *  **            **   * *** * *

A. pisum-NUC          GTGACATGTGGG------CCGATCTGGAGCAGTCAACCCGGTCAAAG------TTAAGCA
B. tabaci-NUC1        AAACGGTCCGGGCTTCGCCCGAAGTGATTCCAAGCACACCGGAAACGCCAAACGGCGGAA
                            *  ***      ****  **   *     ** * *  ** *          * *

A. pisum-NUC          AAGGAAACGGCACGTCCACACATGTG-----------ATCGTCACCGGTAC---------
B. tabaci-NUC1        CAGGAAACGGCCTCATAACAAGAGTGCCACAAGCGAAAACGTTCTCAATCCAGAAAAGGC
                       **********      ***   ***           * ***   *  * *

A. pisum-NUC          ---GTACGACACATGCACACTGGCCGACGTGGACAACGTCCAACAACCACTGTACCTTGA
B. tabaci-NUC1        GTCTTTCCGCCGGAGGCCAGTGGGCGAAGTGAGGTTCATCAAGCTGATCG-AGATCTGCT
                          * *  *    *  ** *** *** ***     * ** * *         * **

A. pisum-NUC          CCTGCCTGGTGCCATACGCGTGCCGCTGTTCTACTGGAAGCTGCACTACGATG-TGGACG
B. tabaci-NUC1        ACGACCTGGAGCGGCA--CCGGGCGGTGTACGCGAGCCACGAGCTGGACGAGCGGATCCG
                       *  ***** **   *  *  * ** *** *    *  *   **   ****       **

A. pisum-NUC          CGGCGGACGGCATCGTGTACATCGGCCTCAACAACCCGTACAAGGAGATCGACGACAGCG
B. tabaci-NUC1        CGGACGACAGCACAGCGTCCC----GCGGATCAACTTCAAGACGGACATCTTCGACGAGG
                      ***  *** ***  * ** *      *  * ****    * * *** ***  ****   *

A. pisum-NUC          TGTACATATGTCCTAA-----CATATGCCCGGACGGT------TACCATGGTCGCGGTTA
B. tabaci-NUC1        AGCGCATCGACGTGAACGCCCTGTACACGCGGAAGGGGCAGCTGGCGCGGCTGACGGCTC
                       *  ***       **       **  * **** **         *   * *  *** *

A. pisum-NUC          CCAGGGCCGCGATCA-CCGAAACAA--------TAAG----GCAG---AGACCGGACCCG
B. tabaci-NUC1        TCCTGGGCTCGGCCGACCTGGCCAACGAGTACATCCGTCGCGACGACCATTTCCTCGCCC
                       *  ** * **  *  **    ***        *  *    *  *   *   *    **

A. pisum-NUC          GCCGCGATCACCGGAAC------GACCCCGAACCGGACGCCAACGACGGACTCATCTACT
B. tabaci-NUC1        GCGGGCATTTCTCGCCCAAGGCCGACTTCGTCCACGCCGCCGAGCAGCTGGCAACCTTCT
                      ** *  **  *  *  *      ***  **  *  * **** *  *       * ** **

A. pisum-NUC          GCTGTACCAAAAA---------------------------------GTCGTTCGAAGAGG
B. tabaci-NUC1        ACTACGTCAACGTCGCGCCCCAGTGGCAGACCTTCAACGGGGGCAACTGGGTCAAACTCG
                       **    ***                                     * * ** **   *

A. pisum-NUC          TTTACGGTGAGCTGGACCCAATCGTGTACAGACAGCTGATG-----------
B. tabaci-NUC1        AGGATGCTCTGAGGGACCTCATCGTCAAGCGCCCCGGAAAACTCCACGTTTA
                         * * *  *  *****  *****  *  * *     *
```

Supplementary Figure S3. Alignment of *A. pisum NUC* and *B. tabaci NUC1* (Accession number: KX390872.1). Red fonts represent *A. pisum* ds*NUC* sequence.

```
A. pisum-NUC      ATGCATTCAACTGCATTAATTACTGCAGTATGTTTAGTCATTTGTTCGTGTCTTCTTCCG
B. tabaci-NUC2    ------------------GTTGGCGCAGTTTGTAAA-------------GTCTCGTTGAA
                                     **   ***** ***  *             ****  **

A. pisum-NUC      ACCATCAATGCCAAGAAAATCAATTATCAAAATGTTAAAAACGAGGACGACTGTTCGTTA
B. tabaci-NUC2    CAGTAACTTGCCAACGAAGGAGCCTCTTTACTTGATTCATAAGGGCTCTAAGCTGGATTT
                          ******  **      * *  *  ** *  * * * *  * *   *   **

A. pisum-NUC      TGGGTGGCC-------GAAAAAGACGAGCCCAAAATGCCCATAC-CGTTCACGAGCAA-C
B. tabaci-NUC2    CGTATATCCTAAGAGCGCAAAAGGAGCGTCTAAAAGGGACAAAGGCTCATTCGAGCTATC
                   *  *  **       * *****  * * * **** *  ** *  *     ***** * *

A. pisum-NUC      GGCAAGCGGTACGCGATCATCTATCCGAACGCCAAGGG----CCGGCTG---GA------
B. tabaci-NUC2    AGAAAACGA----GGAGCTGGTATTCGCATGCCCGGGAAAAGCCAACAAATTGGCACGAA
                   * ** **      ** *   *** ** * ***  **     **  *     *

A. pisum-NUC      CGTGAAGAAAGGGCGTAGTTTCAAGCTGTCGTGCGCCACAGCCAAGTTCGCGTCGAGCGA
B. tabaci-NUC2    CAACCGAGAATGCTGCAGATTCACACTGTGTTAGTGGAA------------------C--
                  *       ** *  * ** ****  ****  *     *                   *

A. pisum-NUC      GCTGCAACGGAACGGCACCTCCGAAGTGTTGGTCACGTGCG---TCGGCATCGACGTCCT
B. tabaci-NUC2    ----------------------GAGGTTTTCTTTAGGAGCAAAGAAGACTTCTAACATCG
                                        ** ** **  * * * **      * * ** *    *

A. pisum-NUC      CGCGTACCGGGGCCAGACGTACCGGTACGAAGACTTCCAGTGCGACGGCATGCCCAAGTC
B. tabaci-NUC2    AGCAGATCGAGTGCACGAGTAGTGT------------------------AAAAGCAACTC
                   **  * ** *  **   ***  *                         *    *** **

A. pisum-NUC      GGAGCTCCGAGTCACCGACGATCGGTGCCAGCCGGCCAACTACACCGTGGCCGTGGTCGG
B. tabaci-NUC2    TTGAACTAGATTCAGGTAAAAAATGCGCCAAAACTG-----GGACACAGATGAAAATTGG
                          ** ***   *  *   * ****             **   *       * **

A. pisum-NUC      TTTCCAGACGGACCACGCATTCCTCCGGCTGTACGGCATGTGTTTCGACAAGTCGACCAA
B. tabaci-NUC2    ATTTGAGGTTGAACGTCAGCTTTTACCTCTGATGTGGGTGTGTCATGATATCAAAGCAGC
                   **  **   ** *      *  * *  ***    *  *****   ** *      *

A. pisum-NUC      GAACAGCCTGTACACTTGGTACGACGCCCGGGCGCCGTACTACGACAACCACCAGAAGTA
B. tabaci-NUC2    GGACACGATTTTTGTCGAGCACGATATTCCAGCCA--CCATCGGCGGAGCACGTATTTTT
                  * ***   * *       * ****    *  **       *  *   * ***      *

A. pisum-NUC      CAGCAAGCGACCGGCGTTCATTAAGTCCA--AAGAGCTGTACGGCAACACGGACGTGAAC
B. tabaci-NUC2    AAAGCTCGGCCAGACTTTGAGGACGGACCTTCTCATCTTTACGAGGGGATTAACGTAAAA
                   *      * * * * ** *  * *  *      * ** ****     *   **** **

A. pisum-NUC      AGGAAATACACTTTCAAAGAACAGAAAAAAACGGTGGCGAAAATACTCAGATCGGACGAA
B. tabaci-NUC2    AAT-------GTTTATACCCAGA-AATATCAACGAGGTTTATTCAATCAGCTCCTTGGAA
                  *          ***  *   * * ** *  *  * **   *   * **** **    ***

A. pisum-NUC      ---CTTGCGGATAAGTACATAAGGAATGATAACCAACACTCGCTGTCCCGGGGCCATTAT
B. tabaci-NUC2    AGGGTCAAGGAGAGAAATT----TATAAAAGGGACATATTATCTGGCGAGGGGTCATCTT
                      *   *** *   *       *   *      * * *  *** *  **** ***  *

A. pisum-NUC      GCAGCCAAAGCTGACTTCTTCTTTGATTTCGAACAGATTTCAACGTTTTACTATGCGAAC
B. tabaci-NUC2    GCCCCTGACGGAGATTTTCTTTACGGATCCTGGCAATGGTCAACATATTTCTACGTAAAC
                  **  *  * *  ** **  * *  *  * *   **    ***** * ** *** *  ***

A. pisum-NUC      GTAGCCCCACAGTGGCAGATATTCAACGGTGACATGTGGGCCGATCTGGAGCAGTCAACC
B. tabaci-NUC2    ACAGCTCCACAGTGGCAAATCATCAATGCGGGCCATTGGCTGGCACTGGAACGATACCTG
                    *** *********** **  **** *  * *   ***   *  ***** *  *

A. pisum-NUC      CGGTCAAAG-TTAAGCAAAGGAAACGGCACGTCCACACATGTGATCGTCACCGGTACGTA
B. tabaci-NUC2    AGGAAGTTTGCAGAGCAGACTGGAGAGGACCTACAC--ATCATGACTGGAATAGTGGGTG
                   **          **** *    *  * ** * ***  **     *   *   **  **

A. pisum-NUC      ----------CGACACATGCACACTGGCCGACGTGGACAACGTCCAACAACCACTGTACC
B. tabaci-NUC2    TGCTTTCTTTTGAATCTGATTCAGGGGAAAATGTGGAGATTTATTTACAGCCAGATGAGC
                             **  *     **  **   * ***** *       *** ***    * *

A. pisum-NUC      TTGACCTGCCTGGTGCCATACGCGTGCCGCTGTTCTACTGGAAGCTGCACTACGATGTGG
B. tabaci-NUC2    -AGAAGATCCGAGTGCCTGCCGC-----GTTTTTCAAAGTCATACGAAGCGAAGTTAGCG
                    **    **  *****   ***     * * *** *    *  *    * * * *   *
```

```
A. pisum-NUC        ACGCGGCGGACGGCATCGTGTACATCGGCCTCAACAACCC-GTACAAGGAGATCGACGAC
B. tabaci-NUC2      ACCGGGCG------ATCGTTGCAGTTTGCTCGAACAACCCCTTCGAAAAAGTTCCAACTT
                    **  ****      *****     *  **   ********  *  **  ** ** *

A. pisum-NUC        AGCGTGTACATATGTCCTAACATATGCCCGGACGGTTACCATGGTCGCGGTTACCAGGGC
B. tabaci-NUC2      TG----------TGTCAGGACATAGCAGC--GGAGCACCGAT---------------GGC
                     *          ****   *****    *     *   * **               ***

A. pisum-NUC        CGCGATCACCGAAACAATAAGGCAGAGACCGGACCCGGCCGCGATCACCGGAACGACCCC
B. tabaci-NUC2      CGACCTCATGGCA-----------------------------------------------
                    **   ***  * *

A. pisum-NUC        GAACCGGACGCCAACGACGGACTCATCTACTGCTGTACCAAAAAGTCGTTCGAAGAGGTT
B. tabaci-NUC2      -----TGATCATGCCAAGGGTCATATCTACTTTTGCGAGGTGAACGATTTC---------
                          **      * * ** *  *******  **       **    ***

A. pisum-NUC        TACGGTGAGCTGGACCCAATCGTGTACAGACAGCTGATG
B. tabaci-NUC2      --CTCTCAAATGCGAGTGGT-------------------
                      *  * *  **       *
```

Supplementary Figure S4. Alignment of *A. pisum NUC* and *B. tabaci NUC2* (Accession number: KX390873.1). Red fonts represent *A. pisum* ds*NUC* sequence.

```
M. persicae-NUC       ATGTATTCAATGTATTCAATTGCATTCACAGCAGTATGTTTAATCATTTGTTCGTGTCTG
P. maritimus-NUC      -------------------------------------GTTTTCTCATTA-----------
                                                           ****  *****

M. persicae-NUC       CGTCCGACCATTTATGCCAAGGAAATCAATCGACCGGTTAAAAACGACGACGACTGTTCG
P. maritimus-NUC      --AACGATCATGTAC------------------------------CAAAAACCATCATC-
                          *** *** **                                *  *    *  **

M. persicae-NUC       CTATGGGTGGCCGAAAAAAACCAGTCCAAAATGCCCATACCGTTCACGAGCAACGGCAAA
P. maritimus-NUC      ------------------------------------------TTGATGAGTGCAACCCAA
                                                                ** * ***      * **

M. persicae-NUC       CAGTACGCGATCATCTATCCGGATGAAAAGGGCAGGCTGCACGCGGAGAAAGGGCGCAGT
P. maritimus-NUC      AATAACCAAATGCTCTACCCGAGCGAATCCAATACAATCAGAATGCGTACCGGGACCCGG
                       *  **   **  **** ***   ***      *   *      *   *  ***  * *

M. persicae-NUC       TTCAAGCTGTCGTGCGCCACTGCCAAGTTCGCGTCGAGCGAACTGCGGCGGGACGATTCC
P. maritimus-NUC      TTCAAGGTAAGCTGCGGCGATAAAGACTTCAAGAAAAAATTCAAAAAATCACCAAGAACC
                      ****** *    **** *  *    * ***  *   *                     **

M. persicae-NUC       TCCGACGCGTTGGTCACGTGCGTCGGCGGCGACGTGCTCGCGTACCGAGGCCAGACGTAC
P. maritimus-NUC      AAAGAAGTCCAAGCACGTTGCAATTCCAAAGACATAATCAATGTCGAAGGCGAACGAATA
                         ** *     *     ***     *   *** *  **     *  **** *

M. persicae-NUC       CGGTACGCCGCCTTCCAGTGCGACGGCATGCCCAAGTCGGAGCTCCGCGTCACCGACGAA
P. maritimus-NUC      CGTTTTCGAGAACTAGAATGTCAAAGTTTTCCAACATCCAAACCCCAAAAAAGAGAAAAT
                      ** *     *   *  * **  *  *  * ** *  **  * * **     *  **  *

M. persicae-NUC       GTGTGCCAGCCGGCCAACTACACCGTGGCCGTGGTCGGTTTCCGGACGGATCGCGCGTTC
P. maritimus-NUC      AAAAAATGCCATGGAAACAACACGCTCTTCGATATAGGATTTCCAACCCGCGACAATTTT
                               *  *  *** ****  *   **   * ** ** *  **      *   **

M. persicae-NUC       CTCCGGCTGTACGGCATGTGCTTCGACAAGTCGACCAAGAACAGCCTGTACACTTGGTAC
P. maritimus-NUC      TTGGATATGATTCGAGTATGTTTCGATGAACTACAACAGGAGTCTCGTTACACCTGGTAT
                       *     **    *  * ** *****  *        ** *    *  ***** *****

M. persicae-NUC       GACGCCCGGTCGCCGTACT-ACGACAACCACCAGAAGTATAGCAAGCGACCGTCGTTCAA
P. maritimus-NUC      GATTCGTCGATGCTACCAACAGGACATCAGAGTAACGTAGGAAGACCTCGATTCGTCCAC
                      **  *   *  **       * **** *      * ***     * *     **** **

M. persicae-NUC       CAAAACCAAAGAGCTGTACGGCAACACGGACGTGAACAAGAAATACACTTTCAAAGAACA
P. maritimus-NUC      GA-------TAATCTCTACAGGTTCCCGGTCG---ACGAGGTGTACAAGAGTAGTTACCA
                       *         * ** *** *   * *** **   ** **   ****     *   * **

M. persicae-NUC       GAGAAACACGGTGGCGACAATACTCAGATCGGACGAACTTGCGGATAAGTACATAAGGAA
P. maritimus-NUC      GCACGATTGGTTTACCAAATTGTTAAAATCTCGAGAAAAAGCTGATCAATATATCAAAAA
                      *    *   * *  * * * *  * * ***    ***   ** *** * ** ** *  **

M. persicae-NUC       TGATAATAAACACTTCCTGTCCCGGGGCCATTACGCAGCCAAAGTTGACTTTTTCTTTGC
P. maritimus-NUC      CGACGGCGAACATTTTCTGTCCAGAGGGCATCTTACGCCAAAGGCTGATATGGTTTATGG
                       **     **** ** ****** * ** ***    *  * ** * ***  *  * * **

M. persicae-NUC       TTATGAACAAACAGCAACGTTTTACTATGCGAACGTTGCCCCGCAGTGGCAGATCTTCAA
P. maritimus-NUC      ATCGGAACAATCAGCAACTTTCCATTATATCAACGTGGCACCTCAGTGGCAAGGTTTCAA
                       *  ****** ******* **  * ***   ***** ** ** ********    *****

M. persicae-NUC       CGGTGACATGTGGGCCGATCTGGAATCGACTACCCGGTCAAAGTTGAGCAAGGAAAACGG
P. maritimus-NUC      CGGTGGTAATTGGAATAAAGTTGAACAAAGTGCAAGAGAAGAGC---TCGAGAAAAAGGA
                      *****  *  ***    *  * ***   * * *  *   * **     * ** **** *

M. persicae-NUC       CACCAGACACGTGATCGTCACCGGCACGTACGAAGTGTGCACGCTGCCCGACGTGGACGA
P. maritimus-NUC      TAAAAGATACCGTGTGGTAACCGGAACCTACGGAGTGGCCACGCTACCAGACGTAAACAA
                       *  *** **    * ** ***** ** **** ****  ****** ** *****  ** *

M. persicae-NUC       CATCCAACAACCACTGTACCTTGACCTGCCCCGTAGCATACCCGTGCCGCTGCTCTACTG
P. maritimus-NUC      CAACGAACAAGAACTCTACCTCTACGAAGACGAGAACAAAAACCCATTGCTCA-------
                      ** * *****  *** *****  **     *   * ** *  *     ***

M. persicae-NUC       GAAGCTGTACTACGATGTGGACACGGAGGACGGCATCGTGTATATCGGTCTCAACAACCC
P. maritimus-NUC      ------------------------------------------------------------
```

```
M. persicae-NUC      CTACAAGGAGATCGACGACAGCGTGTACATATGTCCTAACACATGTCCGGACGGTTACCA
P. maritimus-NUC     ------------------------------------------------------------

M. persicae-NUC      TGGTCGCGGTTACCAGGGCCGCGATCCCCAAAATCATCCATCAGAGACCGAACCAGACCG
P. maritimus-NUC     ------------------------------------------------------------

M. persicae-NUC      CGGTCACCGGAACGACCCCGAACCGGACGCCAACGACGGGCTCATCTACTGCTGTACCAA
P. maritimus-NUC     ------------------------------------------------------------

M. persicae-NUC      AGAGTCATTCGAAGATGCTTACGGCAAGCTGGACCCAATCGTATACCGACAGCTCATGTG
P. maritimus-NUC     ------------------------------------------------------------

M. persicae-NUC      A
P. maritimus-NUC     -
```

Supplementary Figure S5. Alignment of *M*. *persicae NUC* and *P*. *maritimus NUC*. Red fonts represent *M*. *persicae* ds*NUC* sequence.

```
M. persicae-NUC       ------------------------------------------------------------
B. tabaci-NUC1        GCTGATGAAACCGGAAATGAGGTCCGTGCAGTCAGAGTGGAAGTAGAAGAGGATGATGAG

M. persicae-NUC       ------------------------------------------------------------
B. tabaci-NUC1        AAAACGGCAGAAGTGAAGATGGAAATGGGAATGGGTGAAACCGGAGACGGTTTCCGTACC

M. persicae-NUC       ------------------------------------------------------------
B. tabaci-NUC1        ATGAAGCCCGATCATTTCAGTGGTAAACTCAAAGTTGGTAAGCAGGGAGGAGCCGCGTCG

M. persicae-NUC       ------------------------------------------------------------
B. tabaci-NUC1        ACAACTACGAATGGAGGAATTAAGAATGATTTTGAGCTGAGAAACTTGAAAGTCGTGCCC

M. persicae-NUC       ------------------------------------------------------------
B. tabaci-NUC1        GAAAAAGAACCGACGGATACGGAAGTTGACACGTCCGTTACCGTAAAGGAGAGCGGAGGT

M. persicae-NUC       -------ATGTATTCAATG-----------TATTCAATTGCATTCACAGCAGTA-TGTTT
B. tabaci-NUC1        GACTCGAAGCTCCTAAGTTTGTTCGCCGGTTATCGGATGCGAATACAAGAAGGAGTCCAC
                             *  *  * * *            ***   **   * *   ** ** * *

M. persicae-NUC       AATCATTTGTTCGTGTCTG--------CGTCCGACCATTTATGCCAAGGAAATCAATCGA
B. tabaci-NUC1        AATGTTGCCTTCGACTTGCCTTTAAATTTTAGGAGCACTGGTAGCAGGAAACTCGCTCCT
                      ***  *   ****  *             *  ** ** *  *  ** * ** **  **

M. persicae-NUC       CCG---GTTAAAAACGACGACGACTGTTCGCTATGGGTGGCCGAAAAAAACCAGTCCAAA
B. tabaci-NUC1        CTTGTAGTTGAAAATGTCCGCGCGTTGGGCACATTCACAACCGGAAACAACATGAG----
                      *     *** **** * *  **  *       **      *** *** ***  *

M. persicae-NUC       ATGCCCATACCGTTCACGAGCAACGGCAAACAGTACGCGATCATCTATCCGGATGAAAAG
B. tabaci-NUC1        -TGGTACAAACGGTCGCGTGACG--------CGTGATGCATCAAACATTAGAATAAGACA
                       **     * ** ** ** *            **     ****   **  * ** * *

M. persicae-NUC       GGC-----AGGCTGCACGCGGAGAAAGGGCGCAGTT--------TCAA----------GC
B. tabaci-NUC1        GGAAGAGCAGACACCAATCAGAGACGGAATGAAAATGATACTAATGAAAAAAATGTTTGC
                      **      ** *  **  * ****  *   * *  *        * **          **

M. persicae-NUC       TGTCGTGCGCCACTGCCAAGTTC--------GCGTCGAGCGAACTGCGG----CGGGACG
B. tabaci-NUC1        AACCGGAAGCGACGCCAGCCTTCTCGATGACGGTTACAGCTTAAAGCCGCATGATGCACG
                         **   ** **  *    ***        *  *  ***  *  ** *      * ***

M. persicae-NUC       ATTCCTCCGACGCGTTGGTCACGTGCGTCGGCGGCGACGTGCTCGCGTACCGAGGCCAGA
B. tabaci-NUC1        CTTCACCGGAAACGATGTCAGGCTGACTGAAAGCAGTGCTAGCCTCACTGCGAGTAGCAA
                       ***  * **  ** **      **  *    *  *   *   * *    ****     *

M. persicae-NUC       CGTACCGGTACGCCGCCTTCCAGTGCGACGGCATGCCCAAGTCGGAGCTCCGCGTCA-CC
B. tabaci-NUC1        CATCCGCATCGGTGGAATTACGAA-------CATTAACGGGAACGACGGCGGCTTCATCG
                      * * *   *  *  *  ** *          ***   *  *   **   * ** *** *

M. persicae-NUC       GACGAAGTGTGCCAGCCGGCCAAC-----------TACACCGTGGCCG-TGGTCGG-TTT
B. tabaci-NUC1        GAGGAAGTGTAACCCCTGCCGAAAATGCAGCTAGCTCCGCCGGAAATAGTGTAAGCTTTA
                      ** *******  *  * * * **            * * ***       **   *  **

M. persicae-NUC       CCGGACGGATCGCGCGTTCCTCCGGCTGTACGGCATGTGCTTCGACAAGTCGACCAAGAA
B. tabaci-NUC1        CCGGAAATATTGAAGGTCTAATCGAAAGTACCACCATTCACACTGAAGGTGAACCCAGCT
                      *****   ** *   **     **   ****  *   *    *   * **  *** **

M. persicae-NUC       CAGCCTGTACACTTGGTACGACG---------CCCGGTCGCCGTACTACGACAACCACCA
B. tabaci-NUC1        ATACCTATACTGGTTCGAACCTTTCCGGAGGTGACGCCAGCTTTACTAGAAATGGCGCCG
                         *** ***   *   *                **   **  *****  *    * **

M. persicae-NUC       GAAGTATAGCAAG---------------CGACCGTCGTTCAACAAAACCAAAGAGCTGTA
B. tabaci-NUC1        GCTCCACCGGAAATGGCCTCAGCCTTTCCGAATATAGTGCTAATTCCATCAGGAGCTATA
                      *    *  * **                ***   * ** * *        * ***** **

M. persicae-NUC       CGGCAACACGGACGTGA-ACAAGAAATACACTTTCAAAGAACAGAGAAACACGGTGGCGA
B. tabaci-NUC1        CCGCTTTCGCTCGGAACGGCCACAACTCTGCTGGAAATGCC--CATGACCCGAGTCTCAC
                      * **         *     * * ** *   **   ** *     *  * *   **  *
```

```
M. persicae-NUC       CAATACTCAGATCGGACGAACTTGCGGATAAGTACATAAGG---AATGATAATAAAC---
B. tabaci-NUC1        CGGAAATGACCCCAG----TCTTACCGGAAATGACCCCAGTCTTACCGGAAATGACCCCA
                      *   * * *   * *     *** * *  **  **   **    *  *  *** * *

M. persicae-NUC       -ACTTCCTGTCCCGGGGCCATTACGCAGCCAAAGTTGACTTTTTCTTTGCTTATGAACAA
B. tabaci-NUC1        GTCTTACCGGAAATGACCTCAGTCGTACCGGAAATGGCCCTAGTCTTTCCA------GAG
                        *** * *     *  *     ** * *  ** * * * *  ***** *        *

M. persicae-NUC       ACAGCAACGTTTTACTATGCGAACGTTGCCCCGCAGTGGCAGATCTTCAACGGTGACATG
B. tabaci-NUC1        ATGACTCCAATTTTCCCAGAAATAG------------TGCCAGTCTTCCAAGAAACGGTC
                      *   *  *  *** *   *  *  *             **   ***** * *      *

M. persicae-NUC       TGGGCCGATCTGGAATCGACTACCCGGTCAAAGTTGAGCAAGGAAAACGGCACCAGACAC
B. tabaci-NUC1        CGGGCTTCGCCCGAAGTGATTCCAAGCACACCGGAAACGCCAAACGGCGGAACAGGAAAC
                       ****    *  ***  ** * *  *  **  *   *      *   *** **  ** **

M. persicae-NUC       GTGATCGTCACCGGCACGTACGAAGTGTGCACGCTGC---------------------CC
B. tabaci-NUC1        GGCCTCATAACAAGAGTGCCACAAGCGAAAACGTTCTCAATCCAGAAAAGGCGTCTTTCC
                      *   ** * **  *   *    *** *   *** *                       **

M. persicae-NUC       GACGTGGACGACATCCAACAACCACTGTACCTTGACCTGCCCCGTAGCATACCCGTGCCG
B. tabaci-NUC1        GCCGGAGGCCA-GTGGGCGAAGTGAGGTTCATCAAGCTGATCGAGATCTGCTACGACCTG
                      * **  * * *  *     **     ** * *  * ***  *   * *     **  * *

M. persicae-NUC       CTGCTCTACTGGAAGCTGTACT------ACGATGTGGACA----------CGGAGGACGG
B. tabaci-NUC1        GAGCGGCACCGGGCGGTGTACGCGAGCCACGAGCTGGACGAGCGGATCCGCGGACGACAG
                        **   ** **  * *****       ****  *****           **** *** *

M. persicae-NUC       CATCGTGTATATCGGTCTCAACAACCCCTACAAGGAGATCGACGACAGCGTGTACATATG
B. tabaci-NUC1        CACAGCGTCC----CGCGGATCAACTTCAAGACGGACATCTTCGACGAGGAGCGCATCGA
                      **  * **        *  * ****  * * * *** ***  ****   * *  ***

M. persicae-NUC       TCCTAACA-----------CATGTCCGGACGGTTACCATGGTCGCGGTTACCAGGGCCGC
B. tabaci-NUC1        CGTGAACGCCCTGTACACGCGGAAGGGGCAGCTGGCGCGGCTGACGGCTCTCCTGGGCTC
                          ***            *      **  * *  *   * *  *** *  *  ** * *

M. persicae-NUC       GA------TCCCC---AAAATCATCCATCAGAGA----CCGAACCAGACCGCGGT-----
B. tabaci-NUC1        GGCCGACCTGGCCAACGAGTACATCCGTCGCGACGACCATTTCCTCGCCCGCGGGCATTT
                      *       *  **    *   ***** **              *  * ******

M. persicae-NUC       ----CACCGGAACGACCCCGAACCGGACGCCAACGACGGGCTCATCTACTGCTGTAC---
B. tabaci-NUC1        CTCGCCCAAGGCCGACTTCGTCCACGCCGCCGAGCAGCTGGCAACCTTCTACTACGTCAA
                          * *  *  ****  **  *  * ****  *  *   *   * ** ** **

M. persicae-NUC       ------------------------------------CAAAGAGTCATTCGAAGATGCTTA
B. tabaci-NUC1        CGTCGCGCCCCAGTGGCAGACCTTCAACGGGGGCAACTGGGTCAAACTCGAGGATGCTCT
                                                          *   *    * **** ******

M. persicae-NUC       CGGCAAGCTGGACCCAATCGTATACCGACAGCTCATG-------TGA-
B. tabaci-NUC1        GAGG------GACCTCATCGTCAAGCGCCCCGGAAAACTCCACGTTTA
                        *       ****  *****  * ** *     *         *
```

Supplementary Figure S6. Alignment of *M. persicae NUC* and *B. tabaci NUC1*. Red fonts represent *M. persicae* ds*NUC* sequence.

```
M. persicae-NUC     -------ATGTATTCAATGT----------ATTCAATTGCATTCACAGCAGTATGTTTAA
B. tabaci-NUC2      GTTGGCGCAGTTTGTAAAGTCTCGTTGAACAGTAACTTGCCAACGAAGGAGCCTCTTTAC
                             ** *  ** **          * * * ****   *  ** **  * ****

M. persicae-NUC     TCATTTGTTCGTGTCTG----CGTCCGACCATTTATGCCAAGGAAATCAATCGACCGGTT
B. tabaci-NUC2      TTGATTCATAAGGGCTCTAAGCTGGATTTCGTATATCCTAAGAGCGCAAAAGGAGCGTCT
                    *   **  *   * **     *       * * *** * ***      **  ** **  *

M. persicae-NUC     AAAAACGACGACGACTGTTCGCTATGGGTGGCCGAAAAAAACCAGTCCAAAATGCCCATA
B. tabaci-NUC2      AAAAGGGACAAAGGCT---CATTCGAGCTATCAGAAAACGAGGAGC------TGGTATTC
                    ****  *** * * **   *  *   * *  * *****  *  **       **    *

M. persicae-NUC     CCGTTCACGAGCAACGGCAAACAGTACGCGATCATCTATCCGGATGAAAAGGGCAGGCTG
B. tabaci-NUC2      GCATGCCCGGGAAAAGCCAACAAATTG-------------------------GC---ACG
                     * * * ** * ** * ***  * *                           **     *

M. persicae-NUC     CACGCGGAGAAAGGGCGCAGTTTCAAGCTGTCGTGCGCCACTGCCAAGTTCGCGTCGAGC
B. tabaci-NUC2      AACAACCGAGAATGCTGCAGATTCACACTGTGTTAGTGGAACGAGGTTTTCTTTAGGAGC
                     **       ** *  **** ****  ****  *     *  *     ***     ****

M. persicae-NUC     GAACTGCGGCGGGACGATTCCTCCGACGCGTTGGTCACGTGCGTCGGCGGCGACGTGCTC
B. tabaci-NUC2      AAA-----------------------------GAAGACT----------TCTAACATCGA
                     **                             *   **            * *    *

M. persicae-NUC     GCGTACCGAGGCCAGACGTACCGGTACGCCGCCTTCCAGTGCGACGGCATGCCCAAGTCG
B. tabaci-NUC2      GCAGATCGAGTGCACGAGTAGTGTAA------------------------AAGCAACTCT
                    **  * ****  **   ***  *  *                           *** **

M. persicae-NUC     GAGCTCCGCGTCACCGACGAAGTGTGCCAGCCGGCCAACTACACCGTGGCCGTGGTCGGT
B. tabaci-NUC2      TGAACTAGATTCAGGTAAAAAATGCGCCAAAACTGGGAC---AC--AGATGAAAATTGGA
                           *  ***   *  ** ** ****        **   **   *       * **

M. persicae-NUC     TTCCGGACGGATCGCGCGTTCCTCCGGCTGTACGGCATGTGCTTCGACAAGTCGACCAAG
B. tabaci-NUC2      TTTGAGGTTGAACGTCAGCTTTTACCTCTGATGTGGGTGTGTCATGA--TATCAAAGCA-
                    **   *   ** **   * *  * *  ***    *  ****    **    ** *   *

M. persicae-NUC     AACAGCCTGTACACTTGGTACGACGCCCGGTCGCCGTACTACGACAACCACCAGAAGTAT
B. tabaci-NUC2      ----GCGGACACGATTTTTGTCGAGCACGATATTCCAGCCACCATCGGCGGAGCACGTAT
                        **    **  **  *     ** ** *   *   * ** *    *     * ****

M. persicae-NUC     ----AGCAAGCGACC-GTCGTTCA--ACAAAACCAAAGAGCTGTACGGCAACACGGACGT
B. tabaci-NUC2      TTTTAAAGCTCGGCCAGACTTTGAGGACGGACCTTCTCATCTTTACGAGGGGATTAACGT
                        *     ** ** * * ** *  **  * *     * ** ****     *   ****

M. persicae-NUC     GAACAAGAAATACACTTTCAAAGAACAGAGAAACACGGTGGCGACAATACTCAGATCGGA
B. tabaci-NUC2      AAAAAATGTTTATACCCAGAA-----ATATCAACGAGGTTTATTCAATCAGCTCCTTGGA
                     ** **    ** **    **     * *  ***  ***     ****   *   * ***

M. persicae-NUC     CGAACTTGCGGATAAGTACATAAGGAATGATAATAAACACTTCCTGTCCCGGGGCCATTA
B. tabaci-NUC2      AAGGGTCAAGGAGAGAAATTTATAA----AAGGGACATATTATCTGGCGAGGGGTCATCT
                         *   *** *   *  **       *    * * * *  *** *  **** ***

M. persicae-NUC     CGCAGCCAAAGTTGACTTTTTCTTTGCTTATGAACAAACAGCAACGTTTTACTATGCGAA
B. tabaci-NUC2      TGCCCCTGACGGAGATTTTCTTTACGGATCCTGGCAATGGTCAACATATTTCTACGTAAA
                     **  *  * *  ** *** * *  *  *     ***    **** * ** *** *  **

M. persicae-NUC     CGTTGCCCCGCAGTGGCAGATCTTCAACGGTGACATGTGGGCCGATCTGGAATCGACTAC
B. tabaci-NUC2      CACAGCTCCACAGTGGCAAATCATCAATGCGGGCCATTGGCTGGCACTGGAACGATACCT
                    *   ** ** ******** *** **** *  * *   ***   *  ******

M. persicae-NUC     CCGGTCAAAGTTGAGCAAGGAAAACGGCACCAGACACGTGATCGTCACCGGCACGTACGA
B. tabaci-NUC2      GAGG---AAGTTTGCAGAGCAGACTGGAGAGGACCTACACATCATGACTGGAATAGT--G
                      **   *****     ** * *  **       *     *** * ** ** *

M. persicae-NUC     AGTGTGCACGCTGCCCGACGTGGACGAC------ATCCAACAACCACTGTACCTTGACCT
B. tabaci-NUC2      GGTGTGCTTTCTTTTGAATCTGATTCAGGGGAAAATGTGGAGATTTATTTACAGCCAGAT
                     ******   **     *  **    *       **      *    * ***    *  *

M. persicae-NUC     GCCCCGTAGCATACCCGTGCCGCTGCTCTACTGGAAGCTGTACTACGATGTGGACACGG-
B. tabaci-NUC2      GAG---CAGAAGATCCGAGTGCCTGCCGCGTTTTTCAAAGTCATACGAAGCGAAGTTAGC
                    *      ** * * *** *   ****     *       **  ***** * * *    *
```

```
M. persicae-NUC       -AGGACGGCATCGTGTA-TATCGGTCTCAACAACCCCTACAAGGAGATCGACGACAGCGT
B. tabaci-NUC2        GACCGGGCGATCGTTGCAGTTTGCTCGAACAACCCCTTCGAAAAAGTTCCAACTTTG---
                       *    *  *****      * * **  *  * *** *  **  ** ** *     *

M. persicae-NUC       GTACATATGTCCTAACACATGTCCGGACGGTTACCATGGTCGCGGTTACCAGGGCCGCGA
B. tabaci-NUC2        ---------TGTCAGGACATAGCAGCGGAGCAC-------------------------CGA
                               *   *  ****  * *    *                           ***

M. persicae-NUC       TCCCCAAAATCATCCATCAGAGACCGAACCAGACCGCGGTCACCGGAACGACCCCGAACC
B. tabaci-NUC2        TGGCCGAC-------CTCATGGCATGAT--------------------------------
                      *  ** *         ***  *   **

M. persicae-NUC       GGACGCCAACGACGGGCTCATCTACTGCTGTACCAAAGAGTCATTCGAAGATGCTTACGG
B. tabaci-NUC2        -CATGCCAAGGGTCATATCTACTTTTGCGAGGTGAACGATTTCCTCTCAAATGCGAGTGG
                        * ***** *      **  **  ***      ** ** *   **  * ****    **

M. persicae-NUC       CAAGCTGGACCCAATCGTATACCGACAGCTCATGTGA
B. tabaci-NUC2        T------------------------------------
```

Supplementary Figure S7. Alignment of *M. persicae NUC* and *B. tabaci NUC2*. Red fonts represent *M. persicae* ds*NUC* sequence.

```
B. tabaci-NUC1      GCTGATGAAACCGGAAATGAGGTCCGTGCAGTCAGAGTGGAAGTAGAAGAGGATGATGAG
B. tabaci-NUC2      ---------------------GTTGGCGCA------------------------------
                                         **  * ***

B. tabaci-NUC1      AAAACGGCAGAAGTGAAGATGGAAATGGGAATGGGTGAAACCGGAG-ACGGTTTCCGTAC
B. tabaci-NUC2      -----------------GTTTGTAAAG-----------TCTCGTTGAACAGTAAC-TTGC
                                     * * * ** *              **  * ** **  *  * *

B. tabaci-NUC1      CATGAAGCCCGATCATTTCAGTGGTAAACTCAAAGTTGGTAAGCAGGGAGGAGCCGCGTC
B. tabaci-NUC2      CA------------------------------------------ACGAAGGAGCCTCTTT
                    **                                          * * ******* * *

B. tabaci-NUC1      GACAACTACGAATGGAGGAATTAAGAATGATTTTGAGCTGAGAAACTTGAAAGTCGTGCC
B. tabaci-NUC2      ACTTGATTCATA--------------AGGGCTCTAAGCTGGATTTCGTATATCCTAAGAG
                          * *  *              * *  * * *****     * *  *      *

B. tabaci-NUC1      CGAAAAAGAACCGACGGATACGGAAGTTGACACGTCCGTT---ACCGTAAAGGAGAGCGG
B. tabaci-NUC2      CGCAAAAGGAGCGTCTAAAAGGGACAAAGGCTCATTCGAGCTATCAGAAAA---------
                    ** ***** * ** *  * * ***    * * * * **      * * ***

B. tabaci-NUC1      AGGTGACTCGAAGCTCCTAAGTTTGTTCGCCGGTTATCGGATGCGAATACAAGAAGGAGT
B. tabaci-NUC2      --------CGAG--------------GAGCTGGTATTCGCATGC------CCGG------
                            ***                 ** ***  *** ****        *

B. tabaci-NUC1      CCACAATGTTGCCTTCGACTTGCCTTTAAATTTTAGGAGCACTGGTAGCAGGAAACTCGC
B. tabaci-NUC2      -----GAAAAGCCAACAAATTGGCACGAACA-------ACCGAGAATGCTGCAGATTCAC
                              ***  * * *** *   **          *   *   ** * * * ** *

B. tabaci-NUC1      TCCTCTTGTAGTTGAAAAT-GTCCGCGCGTTGGGCACATTCACAACCGGAAACAACATGA
B. tabaci-NUC2      ACTG-TGTTAGTGGAACGAGGTTT--TCTTTAGGAGCAAAGAAGACTTCTAACATCG--A
                     *   *  **** ***    **     * ** **  **   *  **    **** *   *

B. tabaci-NUC1      GTGGTACAAACGGTCGCGTGACGCGTG-ATGCATCAAACATTAGAA--TAAGACAGGAAG
B. tabaci-NUC2      GCAGATCGAG--TGCACGAGTAGTGTAAAAGCAAC----TCTTGAACTAGATTCAGGTAA
                    *  *  * *     * ** *  * **  * *** *      * ***    *  **** *

B. tabaci-NUC1      AGCAGACACCA-ATCAGAGACGGAATGAAAATGATACTAATGAAAAAAATGTTTGCAACC
B. tabaci-NUC2      AAAATGCGCCAAAACTGGGACA--------------CAGATGAAAATTGGATTTGA----
                    *  *  * *** * * * ***               *  *******     ****

B. tabaci-NUC1      GGAAGCGACGCCAGCCTTCTC---G-ATGACGGTTACAGCTTAAAGCCGCATGATGCACG
B. tabaci-NUC2      -GGTTGAACGTCAGCTTTTACCTCTGATGTGGGT-GTGT----------CATGAT-ATCA
                     *     *** **** **  *     ***  ***               ******   *

B. tabaci-NUC1      CTTCACCGGAAACGATGTCAGGCTGACTGAAAGCAGTGCTAGCCTCACTGCGAGTAGCAA
B. tabaci-NUC2      AAGCAGCGGACACGATTTTTGTCGAGCA---CGATATTCCAGCCAC--------------
                       ** **** ***** *  * *   *     *   * * **** *

B. tabaci-NUC1      CATCCGCATCGGTGGAATTACGAACATTAACGGGAACGACGGCGGCTTCATCGGAGGAAG
B. tabaci-NUC2      ------CATCGG---------------------------------------CGGAGCACG
                          ******                                       ***** * *

B. tabaci-NUC1      TGTAACCCCTGCCGAAAATGCAGCTAGCTCCGCCGGAAATAGTGTAAGCTTTACCG-GAA
B. tabaci-NUC2      TAT----------------TTTTAAAGCTCGGCCAGACTTTGAGGACGGACCTTCTCATC
                    * *                      ***** *** **  * * * * *      *

B. tabaci-NUC1      ATATTGAAGGTCTAATCGAAAGTACCACCATTCACACTGAAGGTGAACCCAGCTATACCT
B. tabaci-NUC2      TTTACGAGGGGATTAACGTAAAAA------------AT--GTTTATACCCAGAAATATCA
                     *   ** **  * * ** **  *             *     *  ******  *** *

B. tabaci-NUC1      ATACT---GGTTCGAACCTTTCCGGAG-------------GTGAC------------GCC
B. tabaci-NUC2      ACGAGGTTTATTCAATCAGCTCCTTGGAAAGGGTCAAGGAGAGAAATTTATAAAAGGGAC
                    *         *** * *   ***   *             * **             * *

B. tabaci-NUC1      AGCTTTACTAGAAATGGCGCCGGCTCCA----------CCGGAAATGGCCTCAGCCTTTC
B. tabaci-NUC2      ATATTAT------CTGGCGAGGGGTCATCTTGCCCCTGACGGAGATTTTCTTTACGGATC
                    *  **         *****  ** **             **** **   **   *   **

B. tabaci-NUC1      CGAATATAGTG---CTAATTCCATCAGGAGCT--ATACCGCTTTCGCTCGGAACGGCCAC
B. tabaci-NUC2      CTGGCAATGGTCAACATATTTCTACGTAAACACAGCTCCACAGTGGCA--------AATC
                    *    *  *     *  *** *  *   * *      ** *  * **            *
```

```
B. tabaci-NUC1      AACTCTGCTGGAAATGCCCATGACCCGAGTCTCACCGGAAATGACCCCAGTCTTACCGGA
B. tabaci-NUC2      ATCAATGCGGG-----CCATTGGCTG-----GCACTGGAA-CGATACCTGA------GGA
                    * *  *** **     **  ** *        *** ****  **  ** *       ***

B. tabaci-NUC1      AATGACCCCAGTCTTACCGGAAATGACCCC---AGTCTTACCGGAAA--------TGACC
B. tabaci-NUC2      AGTT-TGCAGAGCAGACTGGAGAGGACCTACACATCATGACTGGAATAGTGGGTGTGCTT
                    * *    *    *  ** *** * ****     *   * ** ****         **

B. tabaci-NUC1      TCAGTCGTACCGGAAATGGCCCTAGTCTTTCCAGAGATGACTCCAATTTTCCCAGAAATA
B. tabaci-NUC2      TCTTTTGAATCTGATT-----CAGGG-GAAAATGTGGAGATTTATTTACAGCCAGA---T
                    **  * * * * **       *  *        * *  ** *    *    *****

B. tabaci-NUC1      GTGCCAGTCTTCCAAGAAACGGTCCGGGCTTCGCCCGAAGTGATTCCAAGCAC-----AC
B. tabaci-NUC2      GAGCAG------------AAGATCCGAGTGCCTGCCGCG-TTTTTCAAAGTCATACGAAG
                    * **              * * **** *   *  ***   *  *** ***        *

B. tabaci-NUC1      CGGAAACGCCAAACG-GCG----GAACAGGAAACGGCCTCATAACAAGAGTGCCACAAGC
B. tabaci-NUC2      CGAAGTTAGCGACCGGGCGATCGTTGCAGTT-----------TGCTCGAACAACCCCTTC
                    ** *     * * ** ***       ***               *  **    * *   *

B. tabaci-NUC1      GAAAACGTTCTCA------ATCCAGAAAAGGCGTCTTTCCGCCGGAGGCCAGTGGGCGAA
B. tabaci-NUC2      GAAAAAGTTCCAACTTTGTGTCAGGACATAGCAGCGGAGCACCGATGGCCGA--------
                    ***** ****  *       **  ** *  **  *    * ***  ****

B. tabaci-NUC1      GTGAGGTTCATCAAGCTGATCGAGATCTGCTACGACCTGGAGCGGCACCGGGCGGTGTAC
B. tabaci-NUC2      ----------------------------------------------------------CC
                                                                               *

B. tabaci-NUC1      GCGAGCCACGAGCTGGACGAGCGGATCCGCGGACGACAGCACAGCGTCCCGCGGATCAAC
B. tabaci-NUC2      TCATGGCATGATCA--------------------------------TGCCAAGGGTCAT-
                     *  * ** ** *                                 * **  ** ***

B. tabaci-NUC1      TTCAAGACGGACATCTTCGACGAGGAGCGCATCGACGTGAACGCCCTGTACACGCGGAAG
B. tabaci-NUC2      ---------ATCTACTTTTGCGAGGTGA------------A-------------------
                               *  ***   ***** *             *

B. tabaci-NUC1      GGCAGCTGGCGCGGCTGACGGCTCTCCTGGGCTCGGCCGACCTGGCCAACGAGTACATC
B. tabaci-NUC2      ------------------------------------------------------------

B. tabaci-NUC1      CGTCGCGACGACCATTTCCTCGCCC--GCGGGCATTTCTCGCCCAAGGCCGACTTCGTCC
B. tabaci-NUC2      -----------CGATTTCCTCTCAAATGCGAGTGGT------------------------
                               * ******** *    *** *   *

B. tabaci-NUC1      ACGCCGCCGAGCAGCTGGCAACCTTCTACTACGTCAACGTCGCGCCCCAGTGGCAGACCT
B. tabaci-NUC2      ------------------------------------------------------------

B. tabaci-NUC1      TCAACGGGGGCAACTGGGTCAAACTCGAGGATGCTCTGAGGGACCTCATCGTCAAGCGCC
B. tabaci-NUC2      ------------------------------------------------------------

B. tabaci-NUC1      CCGGAAAACTCCACGTTTA
B. tabaci-NUC2      -------------------
```

Supplementary Figure S8. Alignment of *B. tabaci NUC1* and *B. tabaci NUC2*. Red and green fonts represent *B. tabaci* ds*NUC1* and ds*NUC2* sequences.
