# Supplementary material for: Non-Target Effects of dsRNA Molecules in Hemipteran Insects
Source: Genes (Basel). 2021 Mar 12;12(3):407. doi: 10.3390/genes12030407 (PMC8000911; doi:10.3390/genes12030407)
Supplement: Supplementary file 1 [file genes-12-00407-s001.zip › Supplementary_information/Supp_tables_121820_AKA0128.docx]

**Supplementary Table S1. Sequence of the primers used in this study**

| **A) qRT-PCR primers** | | | | |
| --- | --- | --- | --- | --- |
| **Species** | **Gene (Accession number)** | **Primer** | **Amplified base-pair number** | **Primer sequence** |
| *A. pisum* | *NUC* | Forward | 132 | CGGACGAACTTGCGGATAA |
|  |  | Reverse |  | GGGCTACGTTCGCATAGTAAA |
|  | *β-tubulin* (ACYPI001007) | Forward | 137 | CGCCAAGTTCTGGCAAGT |
|  |  | Reverse |  | CACCTTCCGAACGGTTGG |
|  | *RPL32* (ACYPI000074) | Forward | 74 | CAAAGTGATCGTTATGACAAACTCAA |
|  |  | Reverse |  | CGTCTTCGGACTCTGTTGTCAA |
| *M. persicae* | *NUC* | Forward | 118 | GGTTACCATGGTCGCGGTTA |
|  |  | Reverse |  | AGTAGATGAGCCCGTCGTTG |
|  | *β-tubulin* (XM_022309483.1) | Forward | 99 | ATCCGAAGAGCACGGAATCG |
|  |  | Reverse |  | GCAGCAGACCCTTCGTTGTA |
|  | *RPL32 (*XM_022324450.1) | Forward | 112 | CAAGCCCAACTGGCGTAAAC |
|  |  | Reverse |  | GTGTCTGGTTCTCTTGTCGC |
| *P. maritimus* | *NUC* | Forward | 100 | TTCGTCGATGCTACCAACAG |
|  |  | Reverse |  | ACTCTTGTACACCTCGTCGA |
|  | *β-tubulin* (MT187989) | Forward | 64 | GCCGGACCTTTCGGTCAAAT |
|  |  | Reverse |  | AATTCGGCACCTTCGGTGTA |
| *B. tabaci* | *NUC1* | Forward | 133 | GAAACTCGCTCCTCTTGTAGTT |
|  |  | Reverse |  | TGTCTGCTCTTCCTGTCTTATTC |
|  | *NUC2* | Forward | 126 | CGAGTGCACGAGTAGTGTAAA |
|  |  | Reverse |  | CACACCCACATCAGAGGTAAA |
|  | *β-tubulin* (XM_019050004.1) | Forward | 176 | CCTTACAACGCTACCCTGTC |
|  |  | Reverse |  | AGACAGGTTGTAACTCCGGA |
| **B) dsRNA primers** | | | | |
| *A. pisum* | *NUC*  (ACYPI008471) | Forward | 328 | ***TAATACGACTCACTATAGG**ACCTCCGAAGTGTTGGTCAC |
|  |  | Reverse |  | **TAATACGACTCACTATAGG**TGTTGCCGTACAGCTCTTTG |
| *M. persicae* | *NUC*  (MYZPE13164) | Forward | 328 | **TAATACGACTCACTATAGGG**TCCTCCGACGCGTTGGTCAC |
|  |  | Reverse |  | **TAATACGACTCACTATAGGG**TGTTGCCGTACAGCTCTTTG |
| *P. maritimus* | *NUC*  (MT187988.1) | Forward | 250 | **TAATACGACTCACTATAGG**CTTGATGAGTGCAACCCAAA |
|  |  | Reverse |  | **TAATACGACTCACTATAGG**TTTTTGGGGTTTGGATGTTG |
| *B. tabaci* | *NUC1*  (KX390872.1) | Forward | 304 | **TAATACGACTCACTATAGG**CTGAAGGTGAACCCAGCTATAC |
|  |  | Reverse |  | **TAATACGACTCACTATAGG**CTGGAAAGACTAGGGCCATTT |
|  | *NUC2*  (KX390873.1) | Forward | 400 | **TAATACGACTCACTATAGG**GGACGGACCTTCTCATCTTTAC |
|  |  | Reverse |  | **TAATACGACTCACTATAGG**CACTCGGATCTTCTGCTCATC |

*The bold and underlined nucleotides is the T7 promoter sequence.
